# Target Finder of Transcription Factor (TFoTF): a novel tool to predict transcription factor‐targeted genes in cancer

**DOI:** 10.1002/1878-0261.13388

**Published:** 2023-02-11

**Authors:** Fanchen Wang, Xiaolin Xu, Xin Li, Jia Yuan, Xuzhu Gao, Chenglong Wang, Wencai Guan, Guoxiong Xu

**Affiliations:** ^1^ Research Center for Clinical Medicine Jinshan Hospital, Fudan University Shanghai China; ^2^ Department of Oncology, Shanghai Medical College Fudan University Shanghai China; ^3^ Center for Tumor Diagnosis & Therapy Jinshan Hospital, Fudan University Shanghai China

**Keywords:** gene expression, position weight matrices, STAT1, transcriptional regulation, tumorigenesis

## Abstract

Transcription factors (TFs) are key players in the regulation of gene transcription in mammalian cells. Although high‐throughput screening can be used to identify differentially expressed genes between comparable groups, the precision of the corresponding datasets is far from optimal. Here, we establish Target Finder of Transcription Factor (TFoTF), a method for the prediction of TF‐targeted genes from genomic and cancer‐related transcriptomic data. TFoTF can identify potential TF‐targeted genes in large cancer datasets and efficiently estimate correlations between TFs and their targeted genes with a significant level of specificity, sensitivity, and precision. Overall, TFoTF is an easy‐to‐use tool that can be utilized to generate testable hypotheses in the context of cancer research projects.

## Graphical abstract

Transcription factors (TFs) are essential for gene transcription and play an important role in tumorigenesis. To predict TF‐targeted genes, the authors developed an easy‐to‐use tool termed Target Finder of Transcription Factor (TFoTF). This *de novo* tool can overcome the constraint of existing prediction methods for TF‐targeted genes in cancer and can perform predictions with more specificity, sensitivity, precision, and time efficiency.

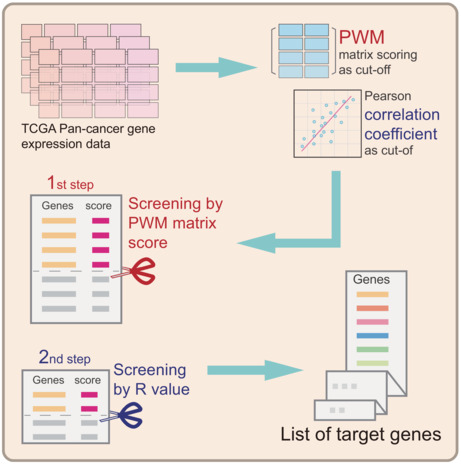

**Abbreviations**
AUC: area under the curve
ChIP‐seq: chromatin immunoprecipitation followed by sequencing
CREB1: cyclic AMP‐responsive element‐binding protein 1
FBS: fetal bovine serum
GO: gene ontology
GTRD: gene transcription regulation database
NGS: next‐generation sequencing
PPI: protein–protein interaction
PWM: position weight matrix
SD: standard deviation
STAT1: signal transducer and activator of transcription 1
SVMs: support vector machines
TCGA: The Cancer Genome Atlas
TF: transcription factor
TFoTF: Target Finder of Transcription Factor
TSS: transcription start site

## 1 Introduction

Transcription factor (TF), a type of DNA‐binding protein, plays an important role in tumorigenesis and progression by regulating target genes at the transcription level. Along with the development and application of high‐throughput screening technologies, numerous TFs are observed to be differentially expressed between cancerous and noncancerous tissues/cells in many studies. Currently, the technology of high‐throughput screening of gene expression is frequently used, allowing for the acquisition of a huge number of differentially expressed genes between comparable groups, and may serve as the foundation for subsequent studies. However, it is currently unclear how accessibility and precision to find the potential TF‐targeted genes for further phenotypic and mechanistic studies are still unsolved [1].

Canonically, TFs bind to the specific region of DNA to regulate target genes, causing the change in gene expression profiles and further leading to the change in cell biological functions [2]. Hence, TFs and target genes are considered to be correlated. To find a TF‐targeted gene to direct the subsequent study, two analytic techniques such as genomic binding data‐based and expression data‐based analyses have currently been facilitated. First, the genomic binding data‐based analyses, including chromatin immunoprecipitation followed by sequencing (ChIP‐seq) and other ChIP‐seq‐based derivative prediction methods such as hTFtarget [3], TFBSTools [4], T‐gene [5], position weight matrix (PWM) [6], etc., identify the genomic‐binding preferences of a given TF, thereby achieving a predictive effect on TF‐targeted genes. ChIP‐seq can provide information from protein‐DNA binding data with some genome coverage [7]. However, the bound sequence, often located within the promoter region of a gene, is a small fraction and may not be sufficient to predict TF‐targeted genes just based solely on the binding domain of the promoter. Second, the expression data‐based analyses may reflect the functions of TFs that regulate their target gene expression and consequently alter the gene profile. Moreover, the regulatory network of TF‐targeted genes can be intervened by the addition of activators or inhibitors. Nevertheless, the expression data‐based analysis has some inherent constraints such as the small sample size and tissue/cell‐specific limitations.

Currently, several other methods have also been used in cancer research. For instance, the TF‐targeted gene predictive algorithm, called T‐Gene [5], can analyze regulatory relationships between TFs and other genes based in part on genomic binding data such as ChIP‐seq. The Support Vector Machines (SVMs) method can predict target genes of specific TFs of *Saccharomyces cerevisiae* in various physiological states [8]. BETA can integrate ChIP‐seq of TFs or chromatin regulators with differential gene expression data to predict TF‐targeted genes [9]. More recently, the gene transcription regulation database (GTRD), an integrated database, can also be used for TF‐targeted gene analysis in knockout, knockdown, or activation experiments [10].

These existing methods, based on different theoretical foundations, provide solutions to a common issue in the study of TF‐targeted gene prediction with encouraging promises. However, there are also some specific shortcomings and limitations for each type of method. For instance, the ChIP‐seq experiment is the core technique based on the analysis of genomic binding data but some constraints of ChIP‐seq limit its practical application in the prediction of TF‐targeted genes. First, the ChIP‐seq is complex and expensive and requires high technical and financial support that some research groups may not be able to afford. Second, the ChIP‐seq data of TFs are multivariate across studies due to tissue and cell specificity. Third, only a limited number of TF‐targeted genes can be identified due to application restrictions although many ChIP‐seq datasets are searchable in public databases. However, the efficacy of expression data‐based analysis is affected by the sample size and experimental performance.

**Fig. 1:**
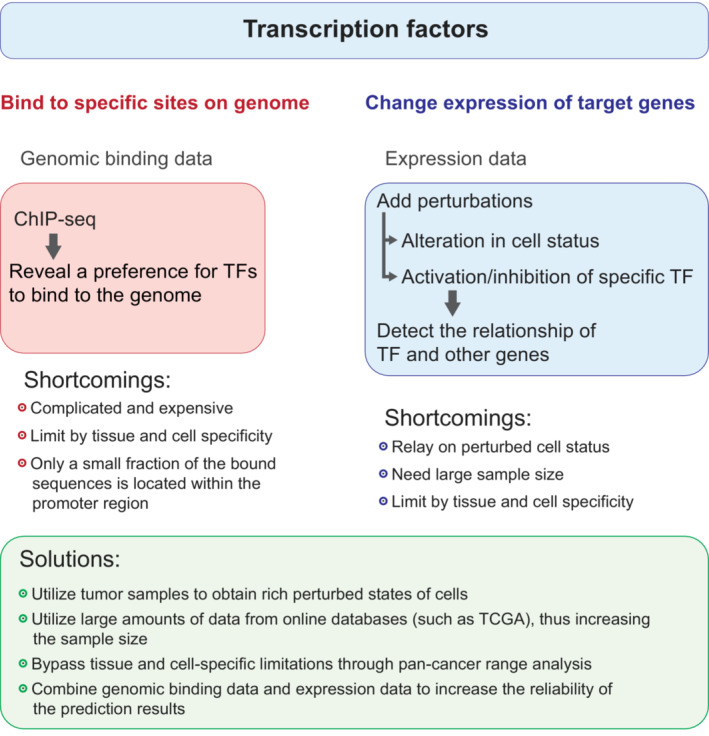
Schematic illustration of strategies for data collection, shortcomings, and current solutions.

Here, we establish a novel tool called Target Finder of Transcription Factor (TFoTF) for the prediction of the potential TF‐targeted genes in large cancer datasets and efficiently estimate correlations between TFs and their target genes. This easy‐to‐use prediction method has been achieved for forecasting targets and may provide practical strategies for TF research in pan‐cancer (Fig. 1).

## 2 Materials and methods

### 2.1 Collection of potential TF‐targeted genes based on pan‐cancer transcriptomic data

Gene expression data in 33 types of cancers were collected from The Cancer Genome Atlas (TCGA) database (https://www.cancer.gov/tcga). The correlation between TF and each gene in individual cancer was analyzed. Maximum (Pearson) correlation coefficients (representing *R*‐scores) of TF and selected genes from all TCGA cancer types were calculated. Genes with *R*‐scores greater than the cut‐off value of a set were selected as candidates.

### 2.2 Evaluation of the newly developed tool compared with ChIP‐seq data

First, we dined TF‐targeted genes from the ChIP‐seq database. The signal transducer and activator of transcription 1 (STAT1), a well‐known TF, was used for practice. ChIP‐seq data for STAT1 were downloaded from Gene Expression Omnibus (GEO, accession # GSM320736; https://www.ncbi.nlm.nih.gov/geo/). The corresponding annotation data were downloaded from the UCSC Genome Browser (NCBI36/hg18; http://genome.ucsc.edu/cgi‐bin/hgTables). ChIP‐seq data were further annotated by the r package chipseeker [11] and genomicfeatures [12] to find STAT1‐targeted genes that were further referred to Ensembl gene ID and those genes that did not exist in the TCGA gene expression datasets were removed. By comparing cancer samples to input DNA control, differentially expressed genes with the ratio of fold change in enrichment > 1.5 and with at least 10 distinct reads were selected as gene targets.

Second, all genes that existed in the TCGA gene expression datasets (denoted by *A*), ChIP‐seq target genes (denoted by *C*) and TFoTF‐predicted genes (denoted by *P*) were compared to get the sensitivity (defined as $\frac{\mathrm{card}\left(C\cap P\right)}{\mathrm{card}\left(C\right)}$) and specificity (defined as $\frac{\mathrm{card}\left(A\right)\;-\;\mathrm{card}\left(C\right)\;-\;\mathrm{card}\left(P\right)\;+\;\mathrm{card}\left(C\cap P\right)}{\mathrm{card}\left(A\right)\;-\;\mathrm{card}\left(C\right)}$) of the method with different cut‐offs. The precision (defined as $\frac{\mathrm{card}\left(C\cap P\right)}{\mathrm{card}\left(P\right)}$) and the relative number of predicted genes (*P*/*C*, defined as $\frac{\mathrm{card}\left(P\right)}{\mathrm{card}\left(C\right)}$) were also calculated, where “card” stands for cardinality, card(*C*) stands for the number of predicted genes, card(*P*) stands for the number of genes in the ChIP‐seq data, “∩” stands for taking the intersection and “*C*∩*P*” stands for the shared genes in ChIP‐seq data and TFoTF‐predicted genes.

### 2.3 Algorithm of TFoTF performed with PWM scoring

Predicted TF‐targeted genes were obtained by an in‐house algorithm (see Data accessibility). Human genome sequence data were downloaded from UCSC Genome Browser (GRCh38/hg38). In general, the promoter region is defined as 100–1000 bp upstream of the transcription start site (TSS) [13]. However, some studies have also pointed out that gene promoters are structurally and functionally complex and the traditional concept of promoter regions can be updated [14]. Therefore, to make the prediction results more comprehensive, we extended the promoter region to 5000 bp upstream from a TSS in each candidate gene. These promoter sequences were extracted from genome sequence data by using the python package biopython (https://biopython.org/). The PWM of each TF was obtained by using r packages JASPAR2020 (JASPAR2020, version 0.99.10; http://jaspar.genereg.net/) and tfbstools [4] and the binding score was calculated. We let **M** denote the PWM scoring matrix and **M** can be expressed as

(1) $$M=\begin{array}{c} A\\ T\\ C\\ G \end{array}\left[\begin{array}{cccc} a_{11} & a_{12} & \cdots & a_{1n}\\ a_{21} & a_{22} & \cdots & a_{2n}\\ a_{31} & a_{32} & \cdots & a_{3n}\\ a_{41} & a_{42} & \cdots & a_{4n} \end{array}\right].$$

For a particular candidate, the promoter sequence of a gene was segmented into a specific length, base by base. For example, if a sequence is “AATTCCGG” and if *n* = 4 (number of bases, stands for the length of TF bind element), the sequence can be segmented into six segments as

(2) $$\text{AATTCCGG}\Rightarrow \begin{array}{c} \text{AATT}\\ \text{ATTC}\\ \text{TTCC}\\ \text{TCCG}\\ \text{CCGG} \end{array}.$$

For one segmented sequence, *x*_1_, *x*_2_, …, *x*_*i*_ represents each base in this segmented sequence and TF can bind to any of these segments. The binding score of one certain base can be expressed as

(3) $$f\left(x_{i}\right)=a_{\mathrm{mn}},\left(m=\left\{\begin{array}{c} 1,x_{i}=A\\ 2,x_{i}=T\\ 3,x_{i}=C\\ 4,x_{i}=G \end{array}\right.,n=i\right).$$

We let *k* denote the total binding score of this segmented sequence and $k=\sum_{1}^{i}\;f\left(x_{i}\right)$. For one promoter sequence, we can get a list of different *k* scores and each score represents the TF‐binding probability of a sequence segment. The highest *k* scores (*k*_max1_ or “max1”), the sum of the top 3 high *k* scores (*k*_max3_ or “max3”), and the sum of all *k* scores (*k*_total_ or “total”) of this promoter sequence were calculated. We collectively referred to these three scores (*k*_max1_, *k*_max3_, *k*_total_) as PWM Score.

### 2.4 Comparison of the possessions of the PWM, ChIP‐seq, hTFtarget, and TFoTF methods

Taking STAT1 as an example, the top 500 targeted genes predicted by the PWM (sort by *k*_max1_), ChIP‐seq (sort by fold‐change), and TFoTF methods were input into PubMed for retrieval. The search query was “(name of predicted gene[Title/Abstract]) AND (STAT1[Title/Abstract])”. If the number of the predicted genes in retrieved results is not zero, those predicted genes are considered as actual TF‐targeted genes defined by that method. The number of relevant literature for each TF‐targeted gene may reflect the quality of a method because that gene had been validated and published. Taking six well‐known TFs (CREB1, SMAD3, SOX2, STAT1, TP53, and ZEB1) as examples, the number of predicted genes was obtained by an open resource hTFtarget. To obtain a more reasonable number of predicted genes, the cut‐off value was set to the 75th percentile of the corresponding score by the TFoTF method. For comparison of the different and common genes in prediction outcomes between hTFtarget and TFoTF, Venn diagrams were plotted.

### 2.5 Web‐based data correction and analysis

Gene Ontology (GO) term analysis was performed using the r packages “clusterprofiler” [15] and “goplot” [16]. Protein–Protein Interaction (PPI) analysis was performed on the website “metascape” with the default setting (https://metascape.org/) [17].

### 2.6 Cell culture, transfection, and qRT‐PCR

Human cell lines used in the study can be identified in Research Resource Identifiers (RRIDs) at https://scicrunch.org/resources. All cell lines were authenticated using Short Tandem Repeat (STR) analysis and checked for mycoplasma‐free. Breast adenocarcinoma (MCF7; RRID:CVCL_0031), colorectal adenocarcinoma (HT‐29; RRID:CVCL_0320), hepatocellular carcinoma (Hep G2; RRID:CVCL_0027), lung carcinoma (A549; RRID:CVCL_0023), prostate carcinoma (DU145; RRID:CVCL_0105), and ovarian cancer (SK‐OV‐3; RRID:CVCL_0532) cells were obtained from American Type Culture Collection (ATCC, Manassas, VA, USA). MCF7, A549, Hep G2, and DU145 cells were cultured in DMEM medium (Sigma‐Aldrich, St. Louis, MO, USA); HT‐29 cells were cultured in RPMI 1640 medium (Sigma‐Aldrich); SK‐OV‐3 cells were cultured in McCoy's 5A medium (Biological Industries, Kibbutz, Israel). All media were supplemented with 10% fetal bovine serum (FBS; Biological Industries). Two siRNAs against STAT1 and cyclic AMP‐responsive element‐binding protein 1 (CREB1) were synthesized from GenePharma (Shanghai, China). To knock down STAT1 or CREB1, cells were transfected with 5 nm STAT1‐siRNA or CREB1‐siRNA for 6 h. After removing transfection reagents, cells were further incubated for 48 h. Total RNA was extracted from cells using the RNA‐Quick Purification kit (ES Science, Shanghai, China). RNA (1 μg) was reversely transcribed using a PrimeScript™ RT Master Mix kit (Perfect Real Time, Takara, Tokyo, Japan). The primers were synthesized from GENEWIZ (Suzhou, China). The sequences of primer and siRNA are listed in Tables S1 and S2, respectively. PCR amplification was performed on the 7300 Real‐Time PCR system (Applied Biosystems, Walthan, MA, USA) using FastStart Universal SYBR Green Master reagent (Roche Diagnostics, Indianapolis, IN, USA). The expression level of target mRNA normalized to a housekeeping gene β‐actin and was calculated by $2^{{\Delta \Delta C}_{t}}$, in which threshold cycle (*C*_t_) was obtained using sequence detection Software V1.4 (Applied Biosystems).

### 2.7 Statistical analysis

Data were analyzed by graphpad prism 9 (GraphPad Software Inc., San Diego, CA, USA). If data follow a normal distribution, an unpaired two‐tailed Student's *t*‐test was applied for comparison between the two groups. If data do not follow a normal distribution, the Kolmogorov–Smirnov test was applied for comparison between the two groups. Results were presented as the mean ± the standard deviation (SD). A *P*‐value of < 0.05 was considered a significant difference.

## 3 Results

### 3.1 Expression correlation between TF and targeted genes at the transcriptomic level is predictable

**Fig. 2:**
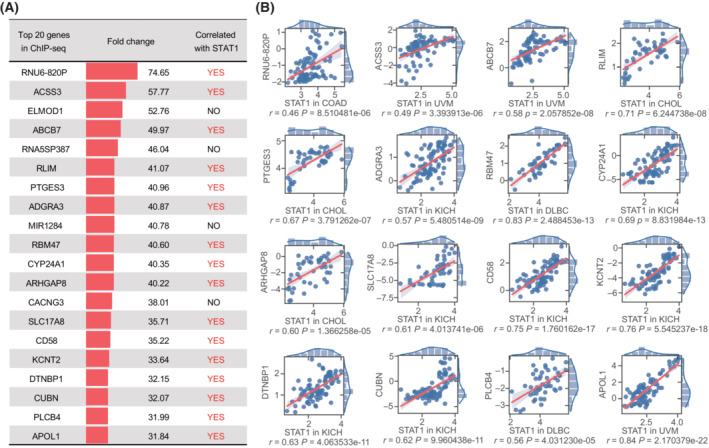
Correlations of STAT1 with gene expression in pan‐cancer. (A) Among the top 20 STAT1‐targeted genes predicted by the Chip‐seq method, 16 genes were correlated with STAT1 at the transcriptomic level in pan‐cancer (Pearson *R* score > 0.45). (B) Scatter plots and linear regression showed the association of STAT1 with the expression of 16 genes detected by the TFoTF method in the corresponding type of cancer (*P* < 0.05). *r* stands for the correlation coefficient; *P* stands for the *P*‐value for a Wald Test with *t*‐distribution.

First, we designed a prediction program for TF‐targeted genes. To demonstrate the predictive capacity and efficiency, we initially selected STAT1, a molecule that has been previously studied in our laboratory, as a representative TF. A series of STAT1‐targeted genes predicted by the ChIP‐seq method were obtained and listed after analyzing the existing STAT1 ChIP‐seq data (GSM320736) from GEO and removing the intergenic match. Second, the top 100 STAT1‐correlated genes were extracted from the TCGA database and listed after analyzing the transcriptomic data in pan‐cancer (Table S3). Further analysis of the top 20 predicted genes in the ChIP‐seq list showed that 16 genes (80%) were correlated with the expression of STAT1 at the transcriptomic level by comparing the two lists and using an appropriate cut‐off value (Fig. 2A,B), suggesting that such approach can be utilized to predict TF‐targeted genes extracted from the TCGA database and that result closed to the outcome of ChIP‐seq experiments.

**Fig. 3:**
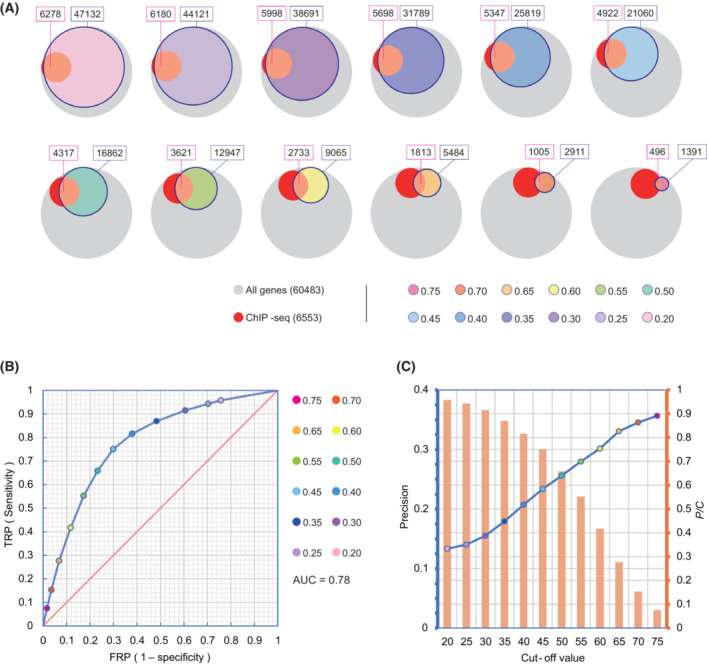
Evaluation of the performance of transcriptomic *R* score‐based target gene prediction. (A) The predicted genes (colored circles with blue line tracing) obtained by changing the cut‐off of the *R* score were marched to TCGA genes (gray circles) and ChIP‐seq genes (red circles). When the *R* score as a cut‐off value was changed (shown by the numbers in the lower right corner), the number of predicted genes (indicated by the area of blue line tracing‐colored circles) was changed subsequently. The Venn diagram reflects the coverage relationship among all genes. (B) Measurement of the sensitivity and specificity. Under the premise of ChIP‐seq as the standard control, the ROC curve of our new method was plotted. The area under the curve (AUC) was calculated to be 0.78. (C) Measurement of precision and number of predicted genes. If the *R* score as a cut‐off value was changed, the precision and the number of predicted genes were changed subsequently. The bar and line chart reflected the changes in precision and the relative number of predicted genes (*P*/*C*) using our new method.

The prediction performance may depend on the choice of the *R*‐score cut‐off. The receiver operating characteristic (ROC) curves between this prediction method and the ChIP‐seq method were compared with the *R*‐score method and a series of outcomes were obtained by changing the cut‐off of the *R*‐score (Fig. 3A). After calculation, the area under the curve (AUC) of our new method was obtained (AUC = 0.78) (Fig. 3B). These results indicate that the prediction method based on the correlation between TF and targeted gene expression has a good prediction performance. In certain circumstances, the precision and the number of predicted genes were preferable to sensitivity and specificity. Therefore, we further calculated the precision and the relative number of predicted genes by matching them to the ChIP‐seq outcomes. As shown in Fig. 3C, the precision of prediction was increased, whereas the relative number of predicted genes was decreased, with the increase in the threshold value. These data suggest that an appropriate threshold value can be chosen based on the experimental purpose and demand in actual needs.

### 3.2 The prediction performance is optimized in combination with PWM scoring

**Fig. 4:**
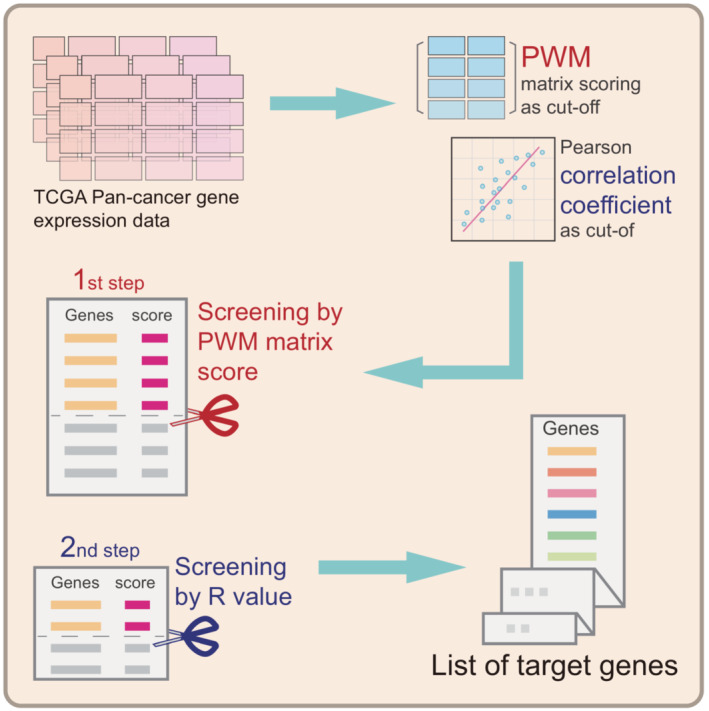
The process diagram of the TFoTF method. The data of gene expression can be downloaded from an online database such as TCGA. The PWM score and the Pearson correlation coefficient *R* score are used as cut‐off values for the next step of screening. By combining PWM scoring with transcriptomic *R* score‐based prediction, a novel algorithm to optimize the precision of prediction is developed. After applying appropriate cut‐off values according to actual needs, a list of predicted TF‐targeted genes can be obtained.

**Fig. 5:**
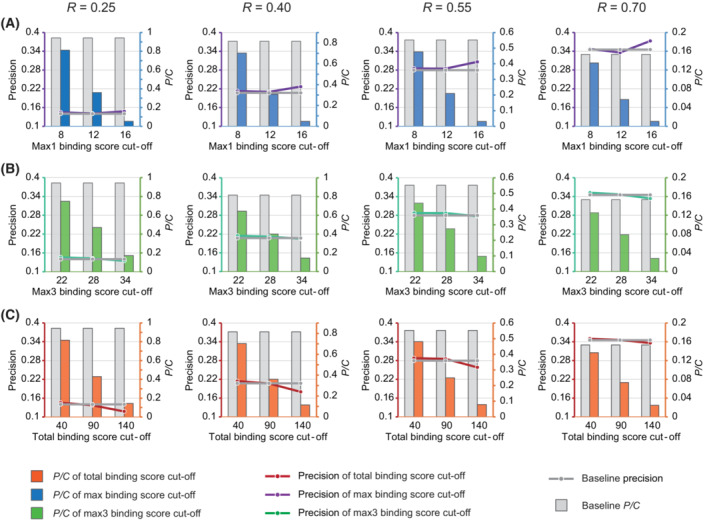
Evaluation of the performance of TFoTF. The prediction efficacy corresponding to different promoter region‐binding scores (PWM scores) and correlation coefficient scores (*R* scores) was calculated. The bar and line charts show the influence of different PWM score cut‐offs, including *k*_max1_ (A), *k*_max3_ (B) and *k*_total_ (C), on precision and a relative number of predicted genes (*P*/*C*) when *R* score cut‐offs were set to 0.25, 0.40, 0.55, and 0.70, respectively.

By combining the existing method of PWM scoring with transcriptomic *R* score‐based prediction, we further developed a novel algorithm to optimize the precision of prediction and named TFoTF (Fig. 4). First, by using STAT1 as an example, the PWM scores, such as *k*_max1_, *k*_max3_ and *k*_total_, of each promoter sequence were calculated and used as evaluation indexes to screen all candidate genes. A list of STAT1 binding scores of the promoter regions in each gene is shown in Table S4. Second, the further screen using *R* score‐based prediction was administered according to the correlation between the expression of each gene and STAT1. After calculating the prediction precision corresponding to different promoter region binding scores and correlation coefficient thresholds, we found that the prediction precision was improved to a certain extent using the *k*_max1_ and the *k*_max3_ scores (Fig. 5A,B; Fig. S1) but was not improved in most cases using the *k*_total_ score (Fig. 5C), indicating that that the *k*_max1_ and *k*_max3_ scores may reflect STAT1‐binding probability and ‐binding preference.

### 3.3 TFoTF is reliable and can be validated

**Fig. 6:**
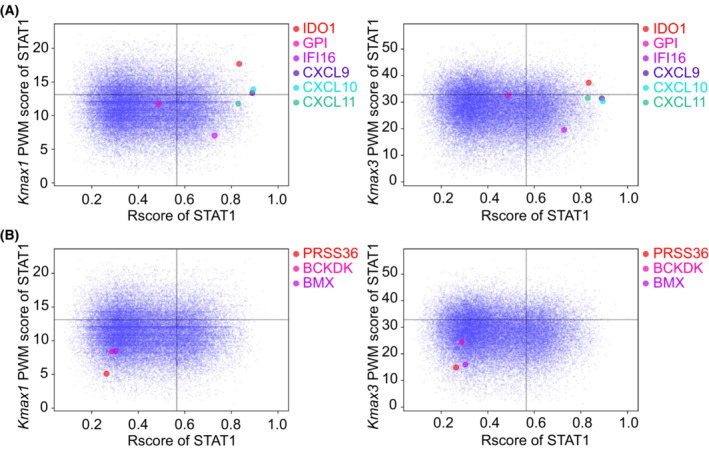
Visualization of predicted outcomes for STAT1‐targeted and nontargeted genes. The *k*_max1_ and *k*_max3_ scores reflected STAT1‐binding probability and ‐binding preference. (A) Prediction of 6 well‐known STAT1‐targeted genes (*IDO1*, *GPI*, *IFI16*, *CXCL9*, *CXCL10*, and *CXCL11*) considered as positive controls. The horizontal axis represents the *R* score of STAT1 for each gene. The vertical axis represents the PWM scores (*k*_max1_ and *k*_max3_) of STAT1 for each gene. (B) Prediction of three STAT1‐non‐targeted genes (*PRSS36*, *BCKKD*, and *BMX*) considered as negatives, in which no STAT1‐binding site in the promoter region of these genes was found. The horizontal axis represents the *R* scores of STAT1 for each gene and the vertical axis represents the PWM scores (*k*_max1_ and *k*_max3_) of STAT1 for each gene. Panels A and B were the same dot plots but the difference between positive and negative genes was distinguishable as shown examples marked.

**Fig. 7:**
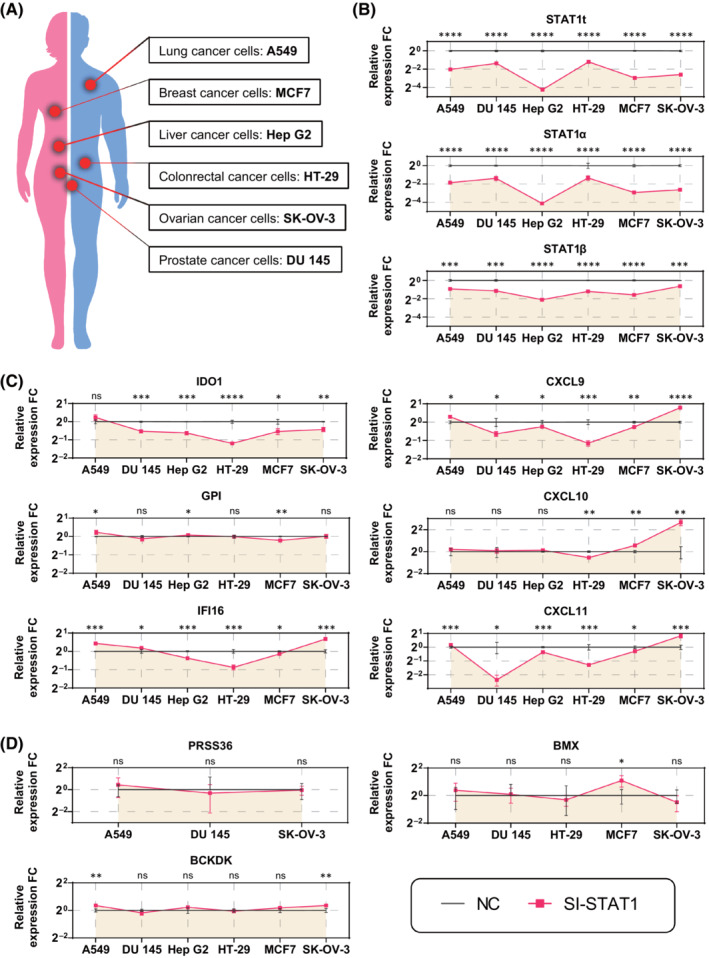
Validation of predicted outcomes by using the loss‐of‐function approach to confirm the regulatory relationship between STAT1 and its target genes in A549, DU145, Hep G2, HT‐29, MCF7, and SK‐OV‐3 cells. (A) Cancer cell lines were derived from different tissue origins. (B) Detection of the knockdown efficiency by STAT1‐siRNA. Total STAT1 (STAT1t) and STAT1α/β isoforms were detected by qRT‐PCR after STAT1 knockdown. (C) Expression levels of IDO1, GPI, IFI16, CXCL9, CXCL10, and CXCL11 were measured by qRT‐PCR after STAT1 knockdown. (D) Expression levels of PRSS36, BCKKD, and BMX were measured by qRT‐PCR after STAT1 knockdown. All results were normalized to β‐Actin before comparison. Each line graph indicates the altered expression levels of the corresponding gene in different cell lines. Horizontal coordinates show different cell lines; vertical coordinates show the relative expression levels of genes. A dark gray dotted line indicates the negative control (NC) group and a red dotted line indicates the STAT1‐siRNA (si‐STAT1) group. The two‐tailed Student's *t*‐test was used for comparison. The “FC” in the vertical axis label represents “fold change”. *n* = 3 independent experiments; ns, no significance; *P < 0.05; **P < 0.01; ***P < 0.001; ****P < 0.0001.

To further prove that TFoTF can precisely predict TF‐targeted genes, we selected six well‐known STAT1‐targeted genes (*IDO1*, *GPI*, *IFI16*, *CXCL9*, *CXCL10*, *CXCL11*) for verification [18]. We found that all six genes had high *R* scores and most of them also had high *k*_max1_ and *k*_max3_ scores (Fig. S2), indicating that these known STAT1‐targeted genes can be precisely identified using this new method as long as an appropriate cut‐off value is selected. The *k*_max1_ and *k*_max3_ scores reflected STAT1‐binding probability and ‐binding preference, where the binding probability was indicated by the PWM score for each binding site in the promoter region (5000 bp upstream from TSS) of each gene. Thus, high *R* scores and high *k*_max1_ and *k*_max3_ scores can be used as the evaluation value to detect above mentioned six known STAT1‐targeted genes that were considered to be positive controls (Fig. 6A; Fig. S3A). For negative controls, three STAT1‐unrelated genes (*PRSS36*, *BCKKD*, *BMX*) that have no STAT1‐binding site in the promoter region were examined. Indeed, there were low *R* scores and low *k*_max1_ and *k*_max3_ scores for these three genes (Fig. 6B; Fig. S3B). Although panels A and B of Fig. 6 were the same dot plots, the difference between positive and negative genes was distinguishable as shown examples marked, suggesting that the STAT1‐binding probability and ‐binding preference can be measured by *k*_max1_ and *k*_max3_ scores. The motif logo and the PWM scores of STAT1 were indicated in Fig. S3C. Using a loss‐of‐function approach by transfecting cells with well‐designed siRNA specific to STAT1, we detected the altered expression levels of these positive and negative control genes in A549, DU145, Hep G2, HT‐29, MCF7, and SK‐OV‐3 cells (Fig. 7; Figs S4–S9). IDO1 was the most significant STAT1‐targeted gene, followed by CXCL10, CXCL9, CXCL11, IFI16, and GPI. As we expected, negative control genes were not regulated by STAT1 knockdown, except BCKDK in A549 and SK‐OV‐3 cells and BMX in MCF7 cells. Furthermore, negative control genes such as PRSS36 in Hep G2, HT‐29, and MCF7 cells and BMX in Hep G2 cells were not detectable (Figs S6–S8). These data indicate the capturing of the regulatory relationship between a TF and its positive, real targeted genes. Thus, TFoTF is a robust prediction tool that can be utilized in various cancers.

**Fig. 8:**
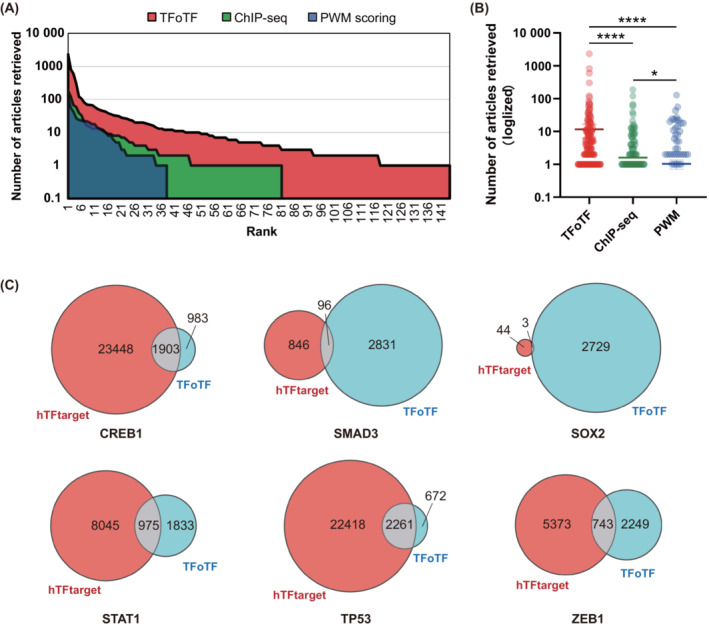
Comparison of different prediction methods. (A) The area plot shows the effect of three different prediction methods (TFoTF, ChIP‐seq, and PWM) in searching STAT1‐targeted genes. The horizontal axis indicates the number of verified genes that exist in the list of the top 500 predicted genes by these three methods. The vertical axis indicates the number of articles retrieved from PubMed for each of these verified genes. (B) The scatter plot reflects differences in the ability of the three methods (TFoTF, ChIP‐seq, and PWM) on predicted target genes. The Kolmogorov–Smirnov test was applied for comparison between groups. *P < 0.05; ****P < 0.0001. (C) Comparison of the predictive effects between TFoTF and hTFtarget. The number of predicted target genes of CREB1, SMAD3, SOX2, STAT1, TP53, and ZEB1 was obtained from an open resource hTFtarget with default setting and TFoTF with the 75th percentile value of the corresponding score as the cut‐off value. Venn diagrams were plotted for the comparison of the different and common genes in predictive outcomes between TFoTF and hTFtarget methods.

Furthermore, the actual performance and efficiency of ChIP‐seq, PWM scoring and our TFoTF were compared. The interactions between TFs and their targeted genes can be evaluated and validated by some published literature [19]. Again, using STAT1 as an example, we performed these three methods for STAT1‐targeted genes and compared those genes after searching for them in PubMed. According to the retrieval data from PubMed, the genes predicted by our TFoTF method exhibited a greater interaction correlation compared to the PWM scoring method (Fig. 8A), suggesting that the TFoTF method performs better for predicting TF‐targeted genes. Moreover, by using pan‐cancer samples, TFoTF overcame the weakness of ChIP‐seq experiments being limited by tissue and cell specificity, providing more comprehensive prediction outcomes (Fig. 8B). Next, we compared our TFoTF to an open resource tool hTFtarget in predicting target genes. Six well‐known TFs (CREB1, SMAD3, SOX2, STAT1, TP53, and ZEB1) were selected. A certain number of different and common targeted genes between the two methods was observed (Fig. 8C). The overlapped genes indicate a valuable prediction, although the number of different genes can be adjusted by the cut‐off value.

### 3.4 Targeted genes of CREB1 are predicted by the TFoTF method and confirmed

**Fig. 9:**
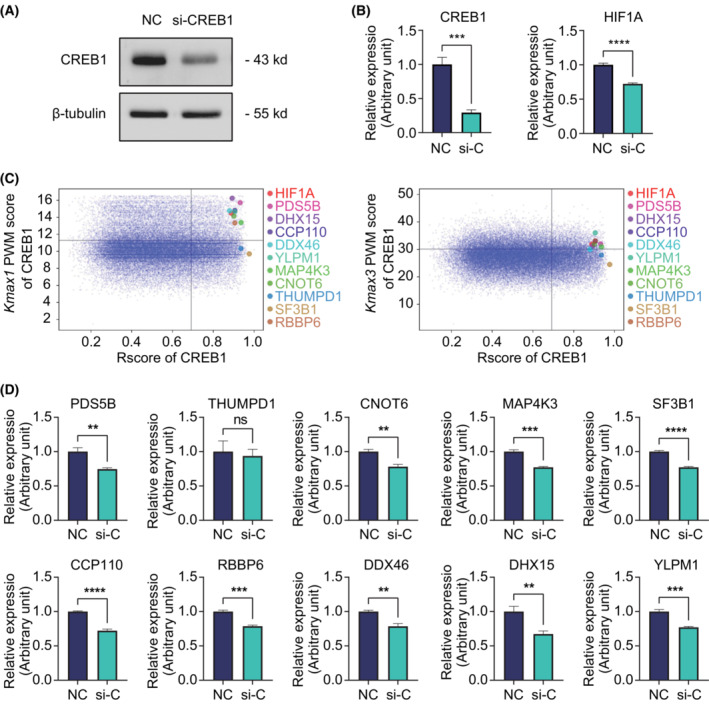
Examination of the prediction efficacy of TFoTF using CREB1 as an example for validation. (A) Knockdown efficiency of CREB1 by siRNA at the protein level was detected by western blot. (B) Knockdown efficiency of *CREB1* and altered expression of *HIF1A*, a known CREB1‐targeted gene, at the mRNA level were detected by qRT‐PCR. The histogram represented the mean ± SD. The two‐tailed Student's *t*‐test was used for comparison. (C) Visualization of predicted genes *HIF1A*, *PDS5B*, *THUMPD1*, *CNOT6*, *MAP4K3*, *SF3B1*, *CCP110*, *RBBP6*, *DDX46*, *DHX15*, and *YLPM1*. The *k*_max1_ (left) and *k*_max3_ (right) scores reflected CREB1‐binding probability and ‐binding preference. The horizontal axis represents the *R* score of CREB1 for each gene. The vertical axis represents the *k*_max1_ and *k*_max3_ PWM scores of CREB1 for each gene. (D) Altered expression levels of *PDS5B*, *THUMPD1*, *CNOT6*, *MAP4K3*, *SF3B1*, *CCP110*, *RBBP6*, *DDX46*, *DHX15*, and *YLPM1* were measured by qRT‐PCT after CREB1 knockdown by CREB1‐siRNA compared to the negative control. The histogram represented the mean ± SD. The two‐tailed Student's *t*‐test was used for comparison. si‐C, si‐CREB1, CREB1‐siRNA; NC, negative control; *n* = 3 independent experiments; ns, no significance; **P < 0.01; ***P < 0.001; ****P < 0.0001.

To confirm our prediction method in practice, we took another TF gene *CREB1* to examine further. CREB1 is a TF that plays an essential role in the regulation of circadian rhythm [20], memory‐related synaptic plasticity [21] and the development of tumors [22]. Again, we performed the TFoTF method to predict and validate the CREB1‐targeted genes using a loss‐of‐function approach. A known‐target gene of CREB1, *HIF1A* [23], was used as a positive control. After the knockdown of CREB1 by siRNA, the expression level of *HIF1A* was significantly decreased (Fig. 9A,B).

According to the transcriptomic data from TCGA, the expression correlation between CREB1 and all 50 793 genes in 33 cancers was calculated using the *R* score. The promoter binding score of CREB1 for all candidate genes was calculated using the PWM (*k*_max_ and *k*_max3_) scoring method (Fig. S10). By taking an *R* score > 0.7 and a *k*_max1_ score > 8 as the cut‐off values, we obtained the predicted genes. To simulate the decision‐making process in real research, we narrowed down a small number of genes with low background expression in ovarian cancer based on the data obtained from the previous study. Compared to *HIF1A* as a positive control, 10 genes (*PDS5B*, *CNOT6*, *MAP4K3*, *SF3B1*, *CCP110*, *RBBP6*, *DDX46*, *DHX15*, *YLPM1*, and *THUMPD1*) from all candidate genes were selected according to our research interests (Fig. 9C). Using qRT‐PCR, we verified the precision of the prediction results after CREB1 knockdown. Among the 10 possible CREB1‐targeted genes, the expression of nine genes (except for *THUMPD1*) was significantly downregulated by CREB1‐siRNA (Fig. 9D). These results indicate that a high‐quality target gene prediction can be achieved by using the TFoTF method we developed.

**Fig. 10:**
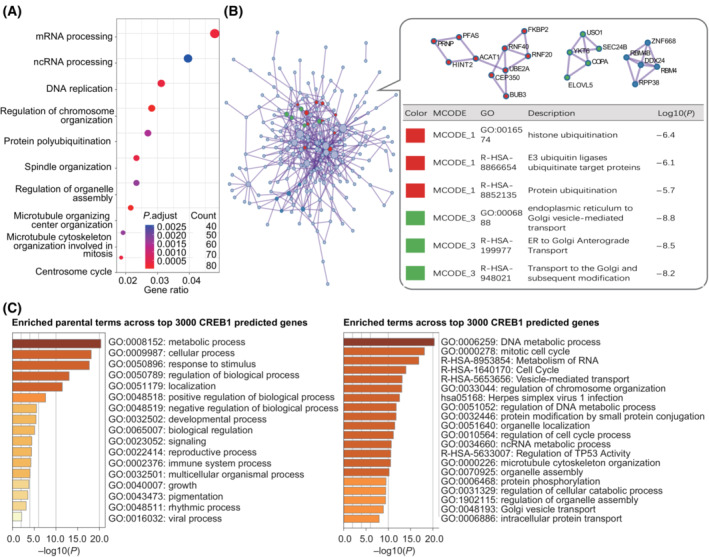
GO/PPI analyses of the prediction of CREB1‐targeted genes. (A) The GO term analysis of the CREB1‐targeted genes predicted by TFoTF. (B) The PPI network analysis. Diagrams were drawn using metascape (https://metascape.org/). Densely connected network components were identified by the Molecular Complex Detection (MCODE) algorithm. Pathway and process enrichment analyses were performed on each MCODE component independently. These MCODE components and the corresponding terms were shown in the bubble box next to the PPI network diagram. (C) The enrichment analysis of the CREB1‐targeted genes predicted by TFoTF. Diagrams were drawn using metascape. The left panel represented enriched parental terms across these genes and the right panel represented more specific GO terms across these genes.

**Fig. 11:**
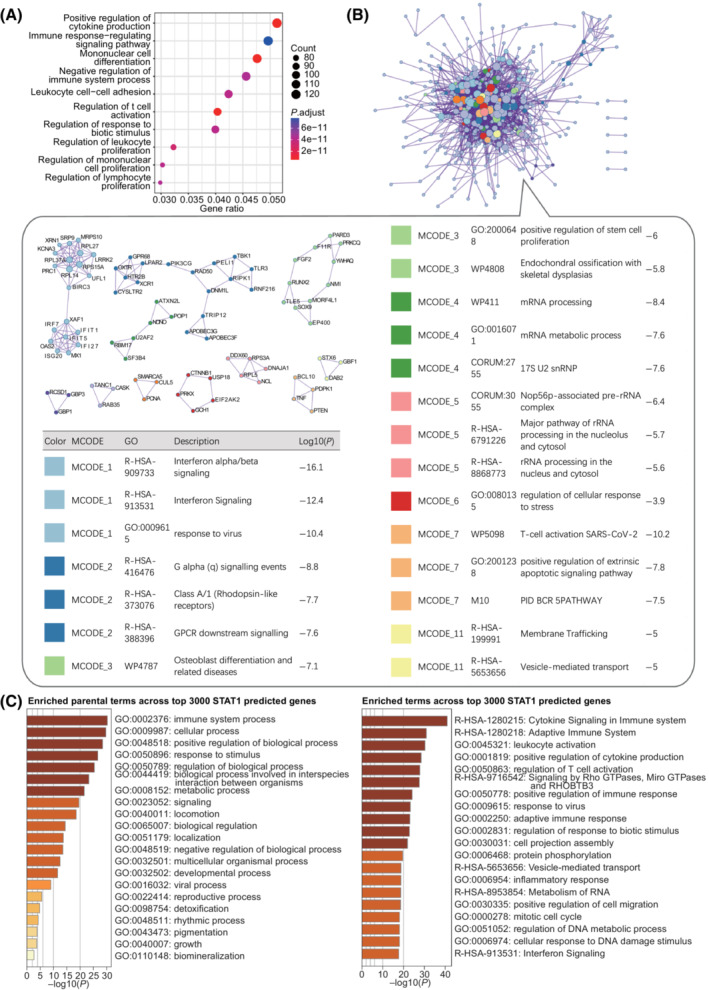
GO/PPI analyses of the prediction of STAT1‐targeted genes. (A) The GO term analysis of the STAT1‐targeted genes predicted by TFoTF. (B) The PPI network analysis. Diagrams were drawn using metascape (https://metascape.org/). Densely connected network components were identified by the Molecular Complex Detection (MCODE) algorithm. Pathway and process enrichment analyses were performed on each MCODE component independently. These MCODE components and the corresponding terms were shown in the bubble box next to the PPI network diagram. (C) The enrichment analysis of the STAT1‐targeted genes predicted by TFoTF. Diagrams were drawn using metascape. The left panel represented enriched parental terms across these genes and the right panel represented more specific GO terms across these genes.

In addition, we performed GO term analysis on the top 3000 genes and PPI analysis on the top 500 genes sorted by the *k*_max1_ scores, respectively. We found that CREB1‐ and STAT1‐targeted genes functioned mainly in the regulation of some specific cellular procession and most of them interacted with each other (Figs 10 and 11). These data suggest that CREB1 and STAT1‐targeted genes predicted by TFoTF tend to be conducive to giving some relatively clearer directions for further study.

## 4 Discussion

The current study established TFoTF for the prediction of TF‐targeted genes from genomic and cancer‐related transcriptomic data. TFoTF was developed as a *de novo* tool that can overcome the constraint of existing prediction methods. TFoTF utilizing expression data on TCGA combined with ChIP‐seq‐sourced TFs binding data may achieve a superior TF‐targeted gene prediction and may provide a strategy for further studies on relationships between TFs and targeted genes.

It has been remarked that the analysis of cancer genomes may rely on variant calling methods [24]. TF is an important regulatory component of the genome. The previous existing methods of TF‐targeted gene prediction can be simply classified into two categories: genomic binding data‐based analysis and expression data‐based analysis. Practically, the problem of poor reliability of genomic binding data‐based analysis can be solved to some extent by combining it with expression data‐based analysis. However, the expression data‐based analysis itself cannot solve some inherent drawbacks such as the small sample size and tissue‐/cell‐specific limitations. Small sample sizes are unreliable for the prediction from data analyses, whereas one type of tissue or cell line is less representative. Therefore, there is an advantage of using the mega data of large cancer samples derived from clinical cases that have existed in public repositories and multiple tissues from pan‐cancer. The TCGA program can be accessible and provide different transcriptomic profiles which can allow us to analyze the correlation extensively between a TF and other expressed genes. Furthermore, we can perform TF‐targeted gene prediction from pan‐cancer transcriptomic profiles to overcome tissue and cell‐specific limitations.

Based on the aforementioned analyses and methods and based on the idea of prediction optimization, we developed TFoTF which is theoretically possible to find the downstream target gene for a particular TF. Indeed, the combined genomic binding data‐based analysis and expression data‐based analysis in pan‐cancer resulted in improved predictions. By comparing with the traditional prediction method such as PWM for predicting a particular TF‐targeted gene based on a score of TF‐binding sequence preference [25], our TFoTF method can further deliver the functional information of candidate genes. Taking CREB1 as an example, the predicted genes tend to be in several similar functional clusters with the given TF. Using six well‐known TFs (CREB1, SMAD3, SOX2, STAT1, TP53, and ZEB1) as further examples, we compared TFoTF with the open resource tool hTFtarget. Although the number of predicted target genes may vary, a certain amount of genes overlapped. Given the difference in design logic between TFoTF and hTFtarget, different functionality may exist. TFoTF enables investigators to better understand the predicted results and may direct them to subsequent studies for gene regulation in cancer research.

The number of predicted genes can be variable because the threshold of a cut‐off value can be set differently depending on the user's requirements, for example, more candidates with less precision or fewer candidates with more precision. Furthermore, a particular TF could bind to its target genes at one or more TF‐binding sites (TFBSs). However, the presence of one or more TFBSs on a gene may not reflect a low‐ or high‐binding affinity with a TF. The advantage of TFoTF is to measure the scores that can reflect TF‐binding probabilities and ‐binding preferences for each binding site in the promoter region, where the relationship between a TF and its targeted genes can be confirmed by ChIP‐seq validation data and gene expression profiles from online resources such as hTFtarget, TCGA, etc. For these unknown target genes (unpublished targets users might discover), researchers may wish to validate them further using their laboratorial experiments.

In practice, the different study objectives often require different prediction properties. For example, some studies may set up low cut‐offs to obtain more candidates and reduce the incidence of false negatives, whereas others may set up higher cut‐offs to obtain fewer candidates and reduce the incidence of false positives. For this reason, we supplied a feasible method in which a certain optimal cut‐off value was not given. To accommodate these different needs, we suggest that researchers can flexibly set up a cut‐off by adjusting the threshold values to obtain a list of target genes that best meet their needs according to the research purpose.

The binding and regulatory effects of TFs on their target genes depend not only on the sequences of the genome but also on the spatial conformation of DNA as well as on various known or unknown epigenetic modifications at multiple levels. Some limitations existed in the current study and need further exploration. First, due to the complexity and unknown nature of the mechanism, only partially TF/target gene pairs in prediction are validated by laboratorial experiments or confirmed by publications. Second, the present study lacks epigenetic considerations. Third, the predictive outcomes might overestimate, while some underestimate, the TF/target gene pairs and the significance of TFs in tumorigenesis. Fourth, the specific binding and regulatory mechanisms between TFs and their targets are not completely understood and require further investigation.

## 5 Conclusions

Target Finder of Transcription Factor, an easy‐to‐use prediction tool, can identify potential TF‐targeted genes in large cancer datasets and efficiently estimate correlations between TFs and targeted genes with a significant level of specificity, sensitivity, and precision. The *de novo* TFoTF can be utilized to generate testable hypotheses in the context of cancer research projects for a better understanding of TF regulation in pan‐cancer.

## Conflict of interest

The authors declare no conflict of interest.

## Author contributions

FW contributed to module generation, data analyses, experiment performance, figure preparation, and manuscript draft. XX, XL, JY, and CW contributed to module improvement and experiment preparation. XG and WG contributed to siRNA and qPCR primer design. GX contributed to the original idea, data analysis, and manuscript writing. All authors read and approved the final manuscript.

## Supporting information


**Fig. S1.** Distribution of PWM scores of STAT1.
**Fig. S2.** Bar charts of the values of the R score and PWM score (*k*_*max*_ and *k*_*max3*_) of six known STAT1‐targeted genes.
**Fig. S3.** STAT1‐binding probability and ‐binding preference.
**Fig. S4.** Validation of predicted outcomes by using the loss‐of‐function approach to confirm the regulatory relationship between a TF and its target genes in lung carcinoma A549 cells.
**Fig. S5.** Validation of predicted outcomes by using the loss‐of‐function approach to confirm the regulatory relationship between a TF and its target genes in prostate carcinoma DU 145 cells.
**Fig. S6.** Validation of predicted outcomes by using the loss‐of‐function approach to confirm the regulatory relationship between a TF and its target genes in hepatocellular carcinoma Hep G2 cells.
**Fig. S7.** Validation of predicted outcomes by using the loss‐of‐function approach to confirm the regulatory relationship between a TF and its target genes in colorectal adenocarcinoma HT‐29 cells.
**Fig. S8.** Validation of predicted outcomes by using the loss‐of‐function approach to confirm the regulatory relationship between a TF and its target genes in breast adenocarcinoma MCF7 cells.
**Fig. S9.** Validation of predicted outcomes by using the loss‐of‐function approach to confirm the regulatory relationship between a TF and its target genes in ovarian cancer SK‐OV‐3 cells.
**Fig. S10.** Distribution of PWM scores of CREB1.
**Table S1.** Primer sequences used in experiments.
**Table S2.** siRNA sequences used in experiments.
**Table S3.** List of the top 100 genes correlated with STAT1 in pan‐cancer.
**Table S4.** The STAT1 binding score of the top 100 genes sorted by *k*_*max*_.

**Appendix S1.** Tutorial for TFoTF.

## Data Availability

The code of TFoTF is available on the public server GitHub at https://github.com/tuitentouten/TFoTF.git. The code, related data, and a short demo video has been deposited and can be downloaded from the Jinshan Hospital webserver at https://www.jinshanhos.org.cn/list/4051/17619.html. A user's guide Tutorial for TFoTF can be found in Appendix S1.
