# Supplementary material for: Target Finder of Transcription Factor (TFoTF): a novel tool to predict transcription factor‐targeted genes in cancer
Source: Mol Oncol. 2023 Feb 11;17(7):1246–62. doi: 10.1002/1878-0261.13388 (PMC10323881; doi:10.1002/1878-0261.13388)
Supplement: Supplementary file 1 — Fig. S1. Distribution of PWM scores of STAT1. Fig. S2. Bar charts of the values of the R score and PWM score (k max and k max3 ) of six known STAT1‐targeted genes. Fig. S3. STAT1‐binding probability and ‐binding preference. Fig. S4. Validation of predicted outcomes by using the loss‐of‐function approach to confirm the regulatory relationship between a TF and its target genes in lung carcinoma A549 cells. Fig. S5. Validation of predicted outcomes by using the loss‐of‐function approach to confirm the regulatory relationship between a TF and its target genes in prostate carcinoma DU 145 cells. Fig. S6. Validation of predicted outcomes by using the loss‐of‐function approach to confirm the regulatory relationship between a TF and its target genes in hepatocellular carcinoma Hep G2 cells. Fig. S7. Validation of predicted outcomes by using the loss‐of‐function approach to confirm the regulatory relationship between a TF and its target genes in colorectal adenocarcinoma HT‐29 cells. Fig. S8. Validation of predicted outcomes by using the loss‐of‐function approach to confirm the regulatory relationship between a TF and its target genes in breast adenocarcinoma MCF7 cells. Fig. S9. Validation of predicted outcomes by using the loss‐of‐function approach to confirm the regulatory relationship between a TF and its target genes in ovarian cancer SK‐OV‐3 cells. Fig. S10. Distribution of PWM scores of CREB1. Table S1. Primer sequences used in experiments. Table S2. siRNA sequences used in experiments. Table S3. List of the top 100 genes correlated with STAT1 in pan‐cancer. Table S4. The STAT1 binding score of the top 100 genes sorted by k max . [file MOL2-17-1246-s001.pdf]

Supplementary file 2 – Figures and Tables

Target Finder of Transcription Factor (TFoTF): a novel tool to predict transcription factor-targeted genes in cancer

Fanchen Wang[1,2], Xiaolin Xu[1,2], Xin Li[1,2], Jia Yuan[1,2], Xuzhu Gao[1], Chenglong Wang[1], Wencai Guan[1], Guoxiong Xu[1,2,3,*]

[1] Research Center for Clinical Medicine, Jinshan Hospital, Fudan University, Shanghai 201508, China

[2] Department of Oncology, Shanghai Medical College, Fudan University, Shanghai 200032, China

[3] Center for Tumor Diagnosis & Therapy, Jinshan Hospital, Fudan University, Shanghai 201508, China

*** Corresponding author:**

Guoxiong Xu, MD, PhD

Scientist, Professor of Oncology

Research Center for Clinical Medicine

Jinshan Hospital, Fudan University

1508 Longhang Road

Shanghai 201508, P.R. China

Tel: +86-21-34189990

Fax: +86-21-57039502

Email: guoxiong.xu@fudan.edu.cn

ORCID: 0000-0002-9074-8754

1 Supplementary Figures

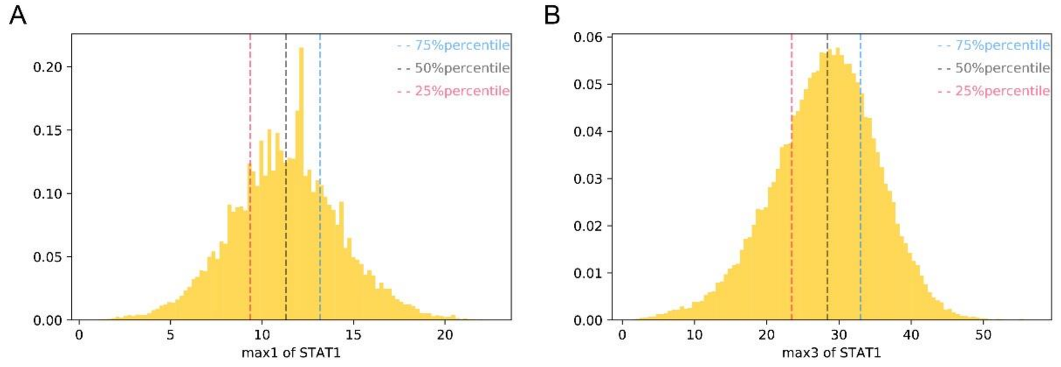

Figure S1. Distribution of PWM scores of STAT1. (A) k_{max1} score. (B) k_{max3} score.

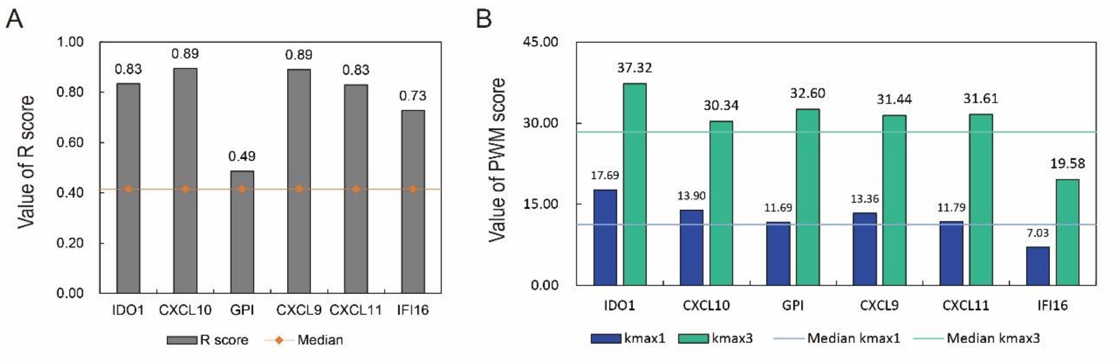

Figure S2. Bar charts of the values of the R score and PWM score (k_{max1} and k_{max3}) of six known STAT1-targeted genes. (A) The orange lines in bar charts indicate the median R score. (B) The values of PWM scores (k_{max1} and k_{max3}) in the STAT1 prediction are shown.

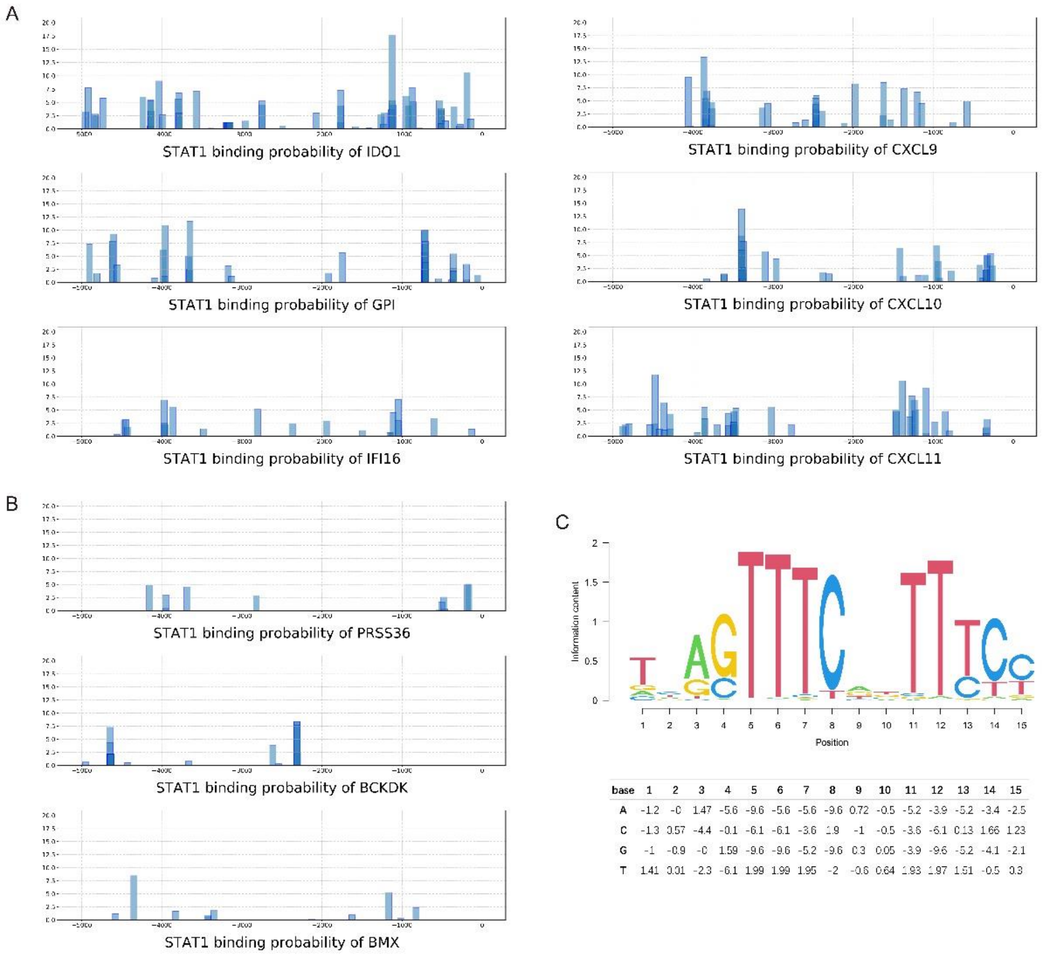

base	1	2	3	4	5	6	7	8	9	10	11	12	13	14	15
A	-1.2	-0	1.47	-5.6	-9.6	-5.6	-5.6	-9.6	0.72	-0.5	-5.2	-3.9	-5.2	-3.4	-2.5
C	-1.3	0.57	-4.4	-0.1	-6.1	-6.1	-3.6	1.9	-1	-0.5	-3.6	-6.1	0.13	1.66	1.23
G	-1	-0.9	-0	1.59	-9.6	-9.6	-5.2	-9.6	0.3	0.05	-3.9	-9.6	-5.2	-4.1	-2.1
T	1.41	0.01	-2.3	-6.1	1.99	1.99	1.95	-2	-0.6	0.64	1.93	1.97	1.51	-0.5	0.3

Figure S3. STAT1 binding probability and binding preference. The binding probability is indicated by the position weight matrices (PWM) score for each binding site in the promoter region (5000 bp upstream from TSS) of each gene. (A) STAT1 binding probability of IDO1, GPI, IFI16, CXCL9, CXCL10, CXCL11. The high scores indicate positive binding. (B) STAT1 binding probability of PRSS36, BCKKD, BMX. The low scores indicate negative binding. (C) The motif logo and the PWM scores of STAT1.

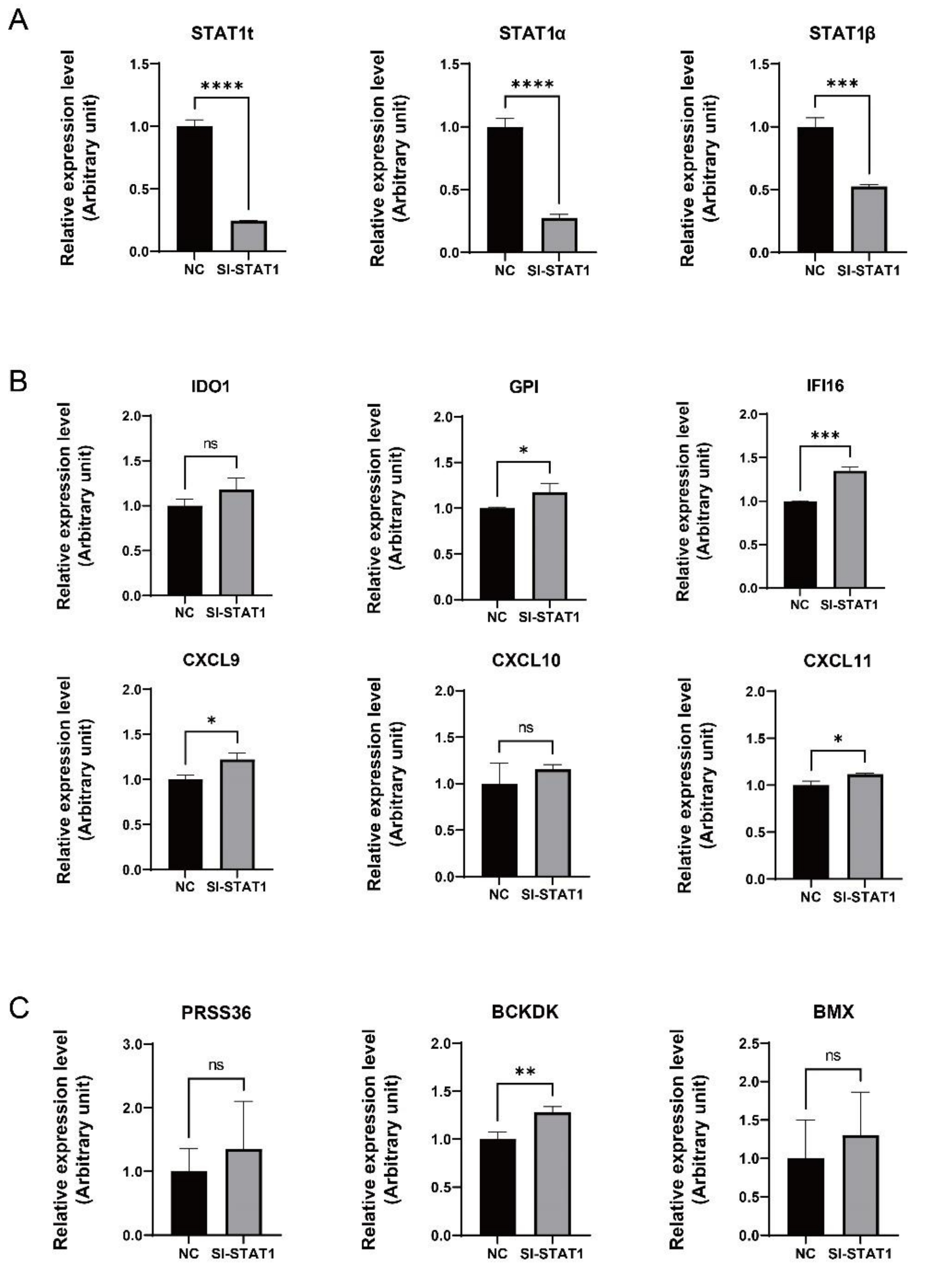

Figure S4. Validation of predicted outcomes by using the loss-of-function approach to confirm the regulatory relationship between a TF and its target genes in lung carcinoma A549 cells. (A) Detection of the knockdown efficiency of STAT1-siRNA. Total STAT1 (STAT1t), and isoforms of STAT1α and STAT1β were detected by qRT-PCR. (B) IDO1, GPI, IFI16, CXCL9, CXCL10, and CXCL11 were detected by qRT-PCR after STAT1 knockdown. (C) Expression of PRSS36, BCKKD, and BMX was detected by qRT-PCR after STAT1 was knocked down. n = 3 independent experiments; ns, no significance; **, P < 0.01; ***, P<0.001; ****, P<0.0001.

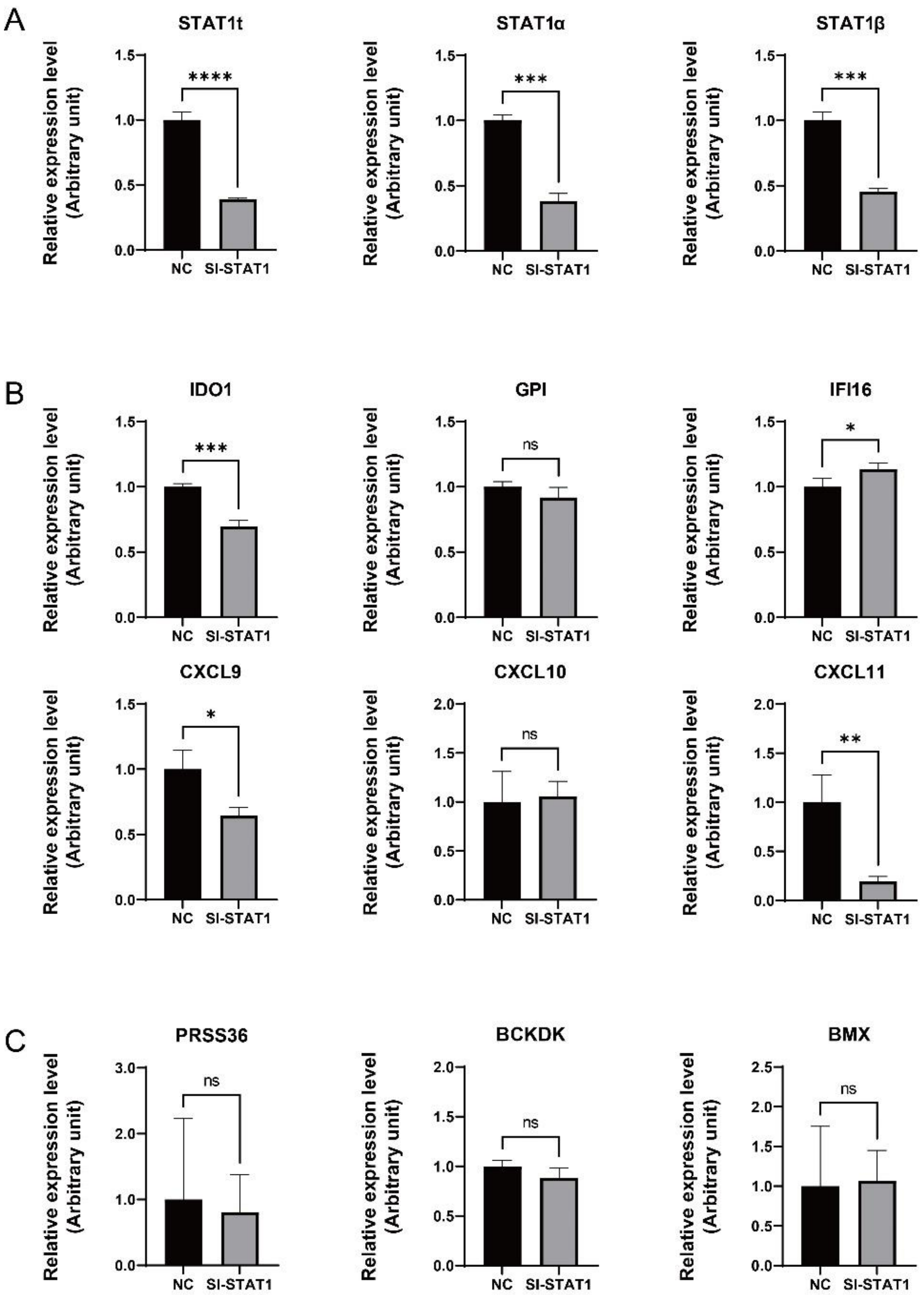

Figure S5. Validation of predicted outcomes by using the loss-of-function approach to confirm the regulatory relationship between a TF and its target genes in prostate carcinoma DU 145 cells. (A) Detection of the knockdown efficiency of STAT1-siRNA. Total STAT1 (STAT1t), and isoforms of STAT1α and STAT1β were detected by qRT-PCR. (B) IDO1, GPI, IFI16, CXCL9, CXCL10, and CXCL11 were detected by qRT-PCR after STAT1 knockdown. (C) Expression of PRSS36, BCKKD, and BMX was detected by qRT-PCR after STAT1 was knocked down. n = 3 independent experiments; ns, no significance; **, $P < 0.01$; ***, $P<0.001$; ****, $P<0.0001$.

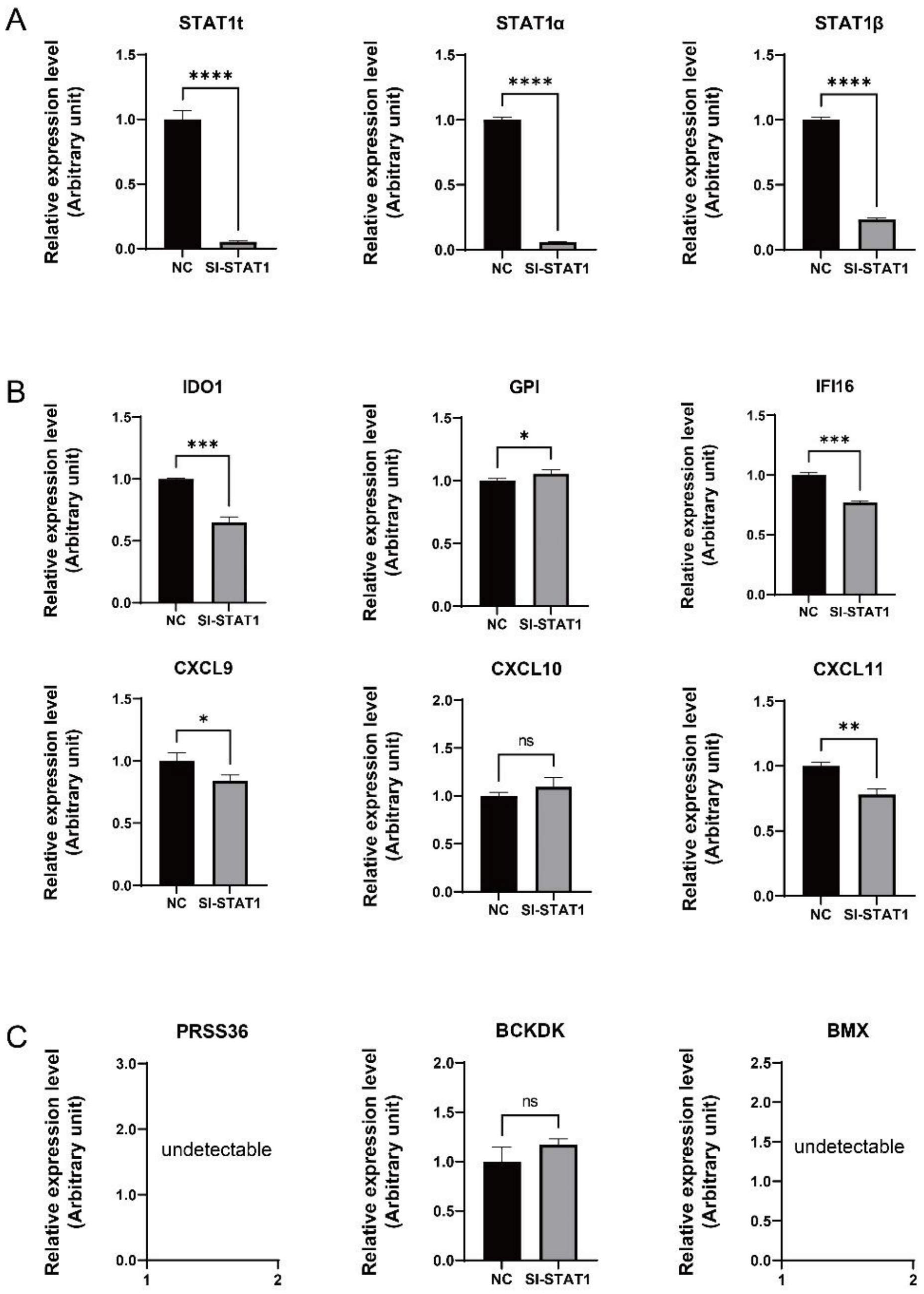

Figure S6. Validation of predicted outcomes by using the loss-of-function approach to confirm the regulatory relationship between a TF and its target genes in hepatocellular carcinoma Hep G2 cells. (A) Detection of the knockdown efficiency of STAT1-siRNA. Total STAT1 (STAT1t), and isoforms of STAT1α and STAT1β were detected by qRT-PCR. (B) IDO1, GPI, IFI16, CXCL9, CXCL10, and CXCL11 were detected by qRT-PCR after STAT1 knockdown. (C) Expression of PRSS36, BCKKD, and BMX was detected by qRT-PCR after STAT1 was knocked down. n = 3 independent experiments; ns, no significance; **, $P < 0.01$; ***, $P<0.001$; ****, $P<0.0001$.

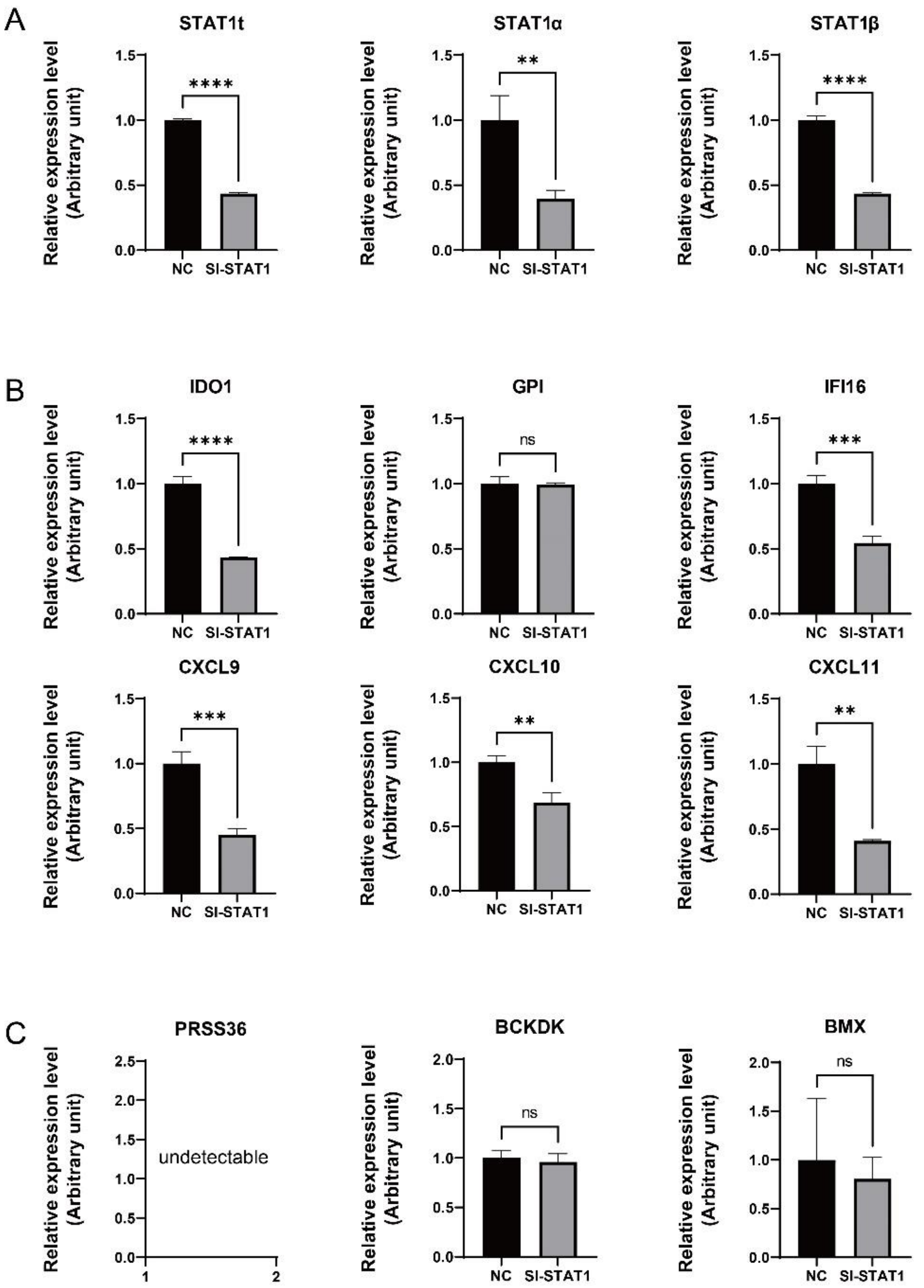

Figure S7. Validation of predicted outcomes by using the loss-of-function approach to confirm the regulatory relationship between a TF and its target genes in colorectal adenocarcinoma HT-29 cells. (A) Detection of the knockdown efficiency of STAT1-siRNA. Total STAT1 (STAT1t), and isoforms of STAT1α and STAT1β were detected by qRT-PCR. (B) IDO1, GPI, IFI16, CXCL9, CXCL10, and CXCL11 were detected by qRT-PCR after STAT1 knockdown. (C) Expression of PRSS36, BCKKD, and BMX was detected by qRT-PCR after STAT1 was knocked down. n = 3 independent experiments; ns, no significance; **, $P < 0.01$; ***, $P<0.001$; ****, $P<0.0001$.

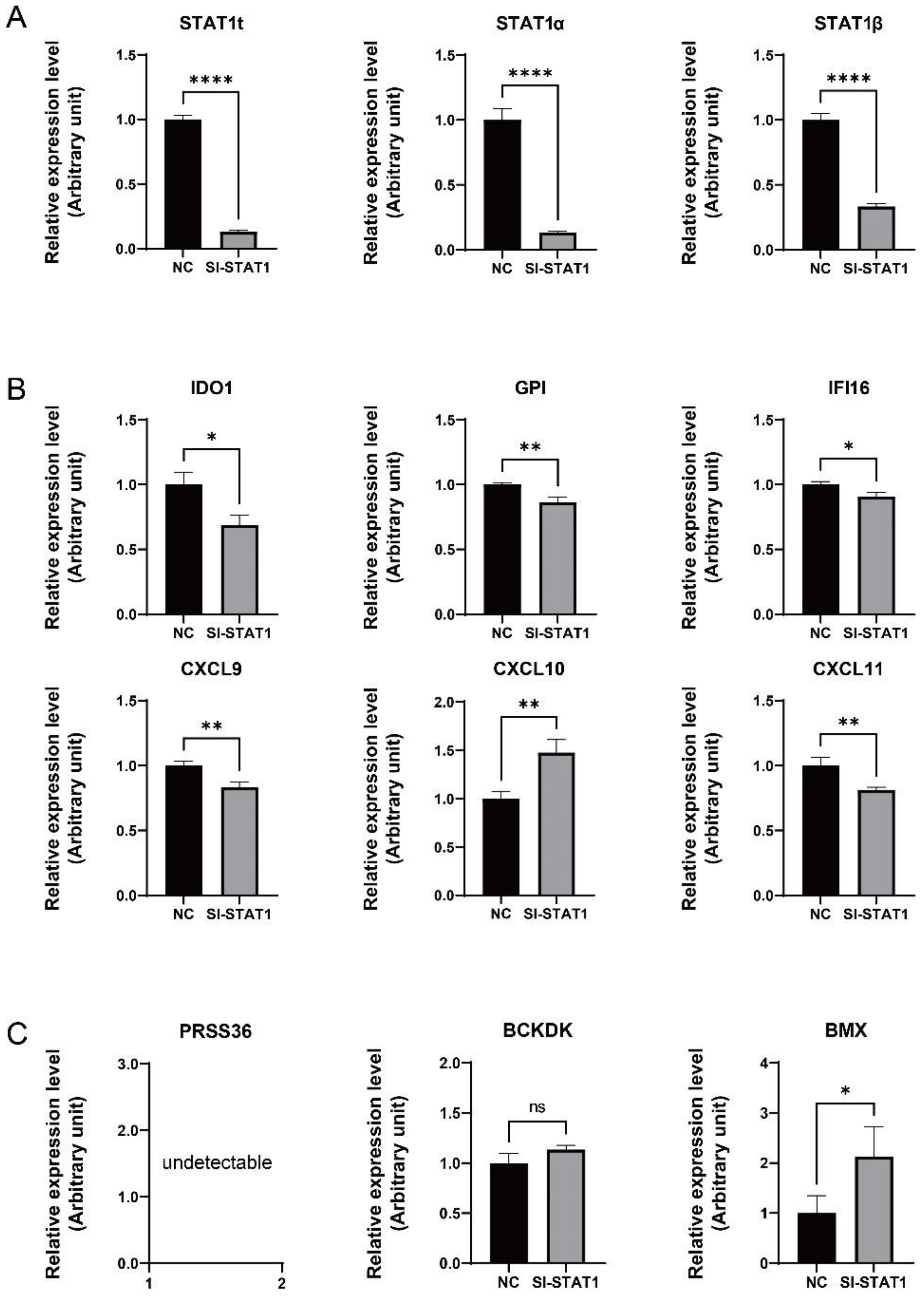

Figure S8. Validation of predicted outcomes by using the loss-of-function approach to confirm the regulatory relationship between a TF and its target genes in breast adenocarcinoma MCF7 cells. (A) Detection of the knockdown efficiency of STAT1-siRNA. Total STAT1 (STAT1t), and isoforms of STAT1α and STAT1β were detected by qRT-PCR. (B) IDO1, GPI, IFI16, CXCL9, CXCL10, and CXCL11 were detected by qRT-PCR after STAT1 knockdown. (C) Expression of PRSS36, BCKKD, and BMX was detected by qRT-PCR after STAT1 was knocked down. n = 3 independent experiments; ns, no significance; **, $P < 0.01$; ***, $P<0.001$; ****, $P<0.0001$.

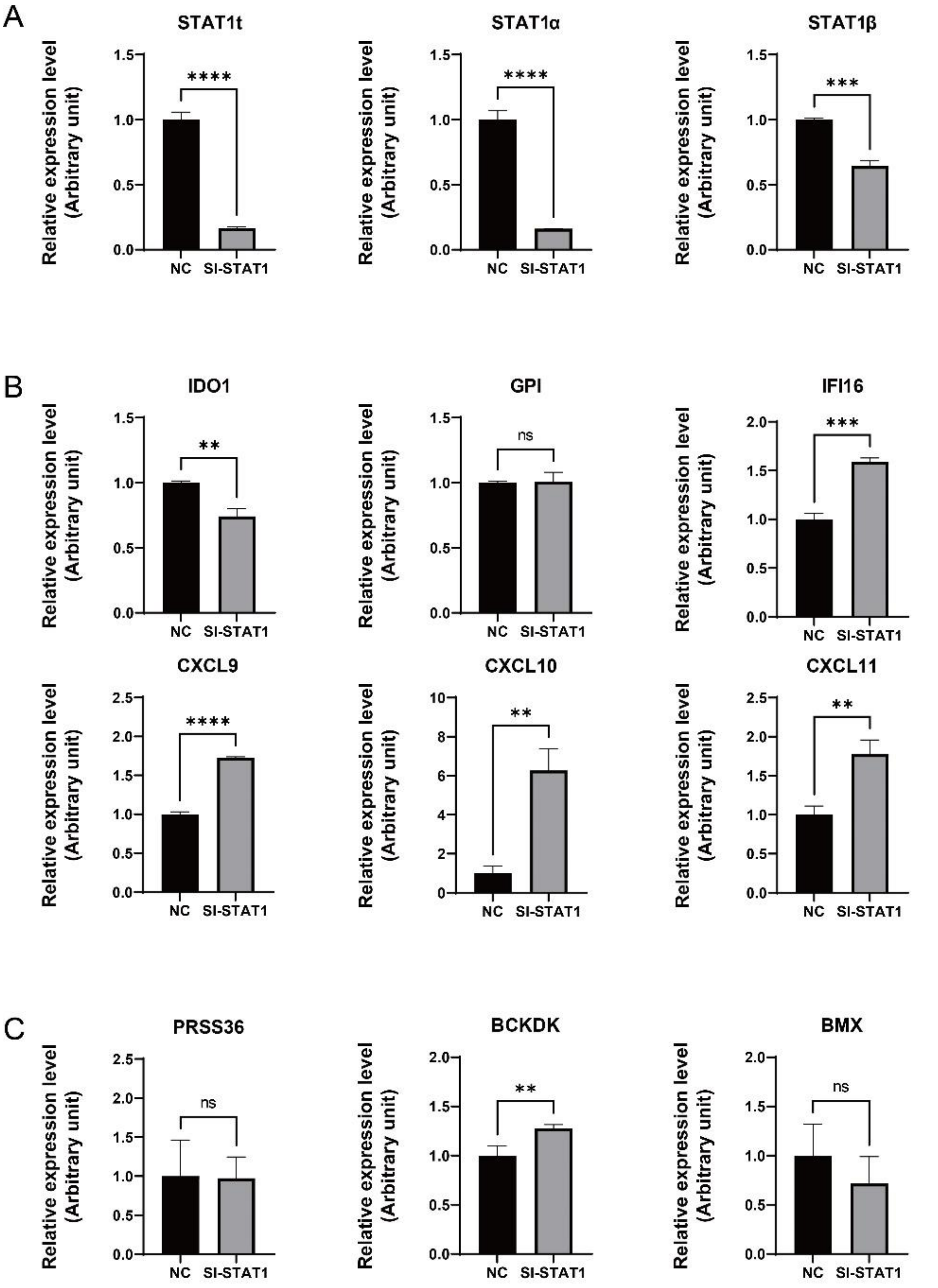

Figure S9. Validation of predicted outcomes by using the loss-of-function approach to confirm the regulatory relationship between a TF and its target genes in ovarian cancer SK-OV-3 cells. (A) Detection of the knockdown efficiency of STAT1-siRNA. Total STAT1 (STAT1t), and isoforms of STAT1α and STAT1β were detected by qRT-PCR. (B) IDO1, GPI, IFI16, CXCL9, CXCL10, and CXCL11 were detected by qRT-PCR after STAT1 knockdown. (C) Expression of PRSS36, BCKKD, and BMX was detected by qRT-PCR after STAT1 was knocked down. n = 3 independent experiments; ns, no significance; **, P < 0.01; ***, P<0.001; ****, P<0.0001.

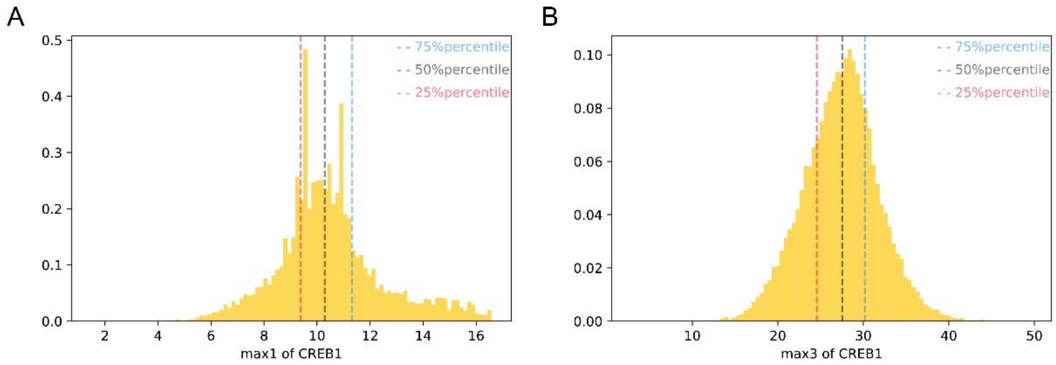

Figure S10. Distribution of PWM scores of CREB1. (A) k_{max1} score. (B) k_{max3} score.

2 Supplementary tables

Table S1. Primer sequences used in experiments.

**qRT-PCR primers**	**Sequence (5' → 3')**	**Position in sequence**	**GenBank Accession #**
CREB1			
Forward	ATTCACAGGAGTCAGTGGATAGT	nt 468-490	NM_004379.5
Reverse	CACCGTTACAGTGGTGATGG	nt 649-630	
Beta-actin			
Forward	TCATCACCATTGGCAATGAG	nt 824-843	NM_001101.5
Reverse	CACTGTGTTGGCGTACAGGT	nt 978-959	
HIF1A			
Forward	GAACGTCGAAAAGAAAAGTCTCG	nt 338-360	NM_001530.4
Reverse	CCTTATCAAGATGCGAACTCACA	nt 461-439	
PDS5B			
Forward	ATCTATCAGCGATTTGTCAGAGC	nt 899-921	NM_015032.4
Reverse	CAAGCTGGGGTAAAACAGAGAG	nt 993-972	
THUMPD1			
Forward	TTTGCGAATGTTACCCATCTCA	nt 556-577	NM_017736.5
Reverse	TCTGAAATGTCCCTTTGTTTGGA	nt 671-649	
CNOT6			
Forward	GACGGCTGCTGAACTATTTGC	nt 827-847	NM_001370472.1
Reverse	GGCCTTGTCCTATCTGGTTCT	nt 941-921	
MAP4K3			
Forward	AACCCCGGCTTCGATTTGTC	nt 302-321	NM_003618.4
Reverse	AACATTCCGTGCCTTGTAGAC	nt 406-386	
SF3B1			
Forward	GTGGGCCTCGATTCTACAGG	nt 120-139	NM_012433.4
Reverse	GATGTCACGTATCCAGCAAATCT	nt 199-177	
CCP110			
Forward	AGACGCAGTCTGAGAGGTAGT	nt 895-915	NM_001199022.3
Reverse	CAGTGTTTGCCTGTCAACTGG	nt 1016-996	
RBBP6			
Forward	ATGACTCTTCCGCGTCTATTTCT	nt 1369-1391	NM_006910.5
Reverse	CCAGATTGCGACATCATTGCTTT	nt 1477-1455	
DDX46			
Forward	AAAATGGCGAGAAGAGCAACG	nt 664-684	NM_001300860.2
Reverse	CATCATCGTCCTCTAAACTCCAC	nt 773-751	
DHX15			
Forward	GGGGACCGATGGGAAGGAT	nt 221-239	NM_001358.3
Reverse	TAGCATTTGTTGAAGCTCGCA	nt 363-343	
YLPM1			
Forward	TCCTCGCAATCCTATTTGAGCC	nt 811-832	NM_019589.3
Reverse	GTGGTGGAGCTAGTAGTTGGG	nt 919-899	

IDO1			
Forward	GCCAGCTTCGAGAAAGAGTTG	nt 190-210	NM_002164.6
Reverse	ATCCCAGAACTAGACGTGCAA	nt 285-265	
CXCL10			
Forward	GTGGCATTCAAGGAGTACCTC	nt 116-136	NM_001565.4
Reverse	TGATGGCCTTCGATTCTGGATT	nt 313-292	
GPI			
Forward	CCGCGTCTGGTATGTCTCC	nt 740-758	NM_001184722.1
Reverse	CCTGGGTAGTAAAGGTCTTGGA	nt 852-831	
CXCL9			
Forward	CCAGTAGTGAGAAAGGGTCGC	nt 130-150	NM_002416.3
Reverse	AGGGCTTGGGGCAAATTGTT	nt 228-209	
CXCL11			
Forward	GACGCTGTCTTTGCATAGGC	nt 166-185	NM_005409.5
Reverse	GGATTTAGGCATCGTTGTCCTTT	nt 313-291	
IFI16			
Forward	GTTTGCCGCAATGGGTTCC	nt 2013-2031	NM_001206567.2
Reverse	ATCTCCATGTTTCGGTCAGCA	nt 2089-2069	
BMX			
Forward	GAGCAGACGCCTGTAGAGAGA	nt 351-371	NM_203281.3
Reverse	ACCCACTATGGTACTTGACCAG	nt 513-492	
PRSS36			
Forward	CTGCTTTCTGGACCCGAACAG	nt 1120-1140	NM_173502.5
Reverse	GTCCCACGAAGCGTTCTCG	nt 1249-1231	
BCKDK			
Forward	GACTTCCCTCCGATCAAGGAC	nt 683-703	NM_005881.4
Reverse	CTCTCACGTAGGCCCTCTG	nt 798-780	

Table S2. siRNA sequences used in experiments.

**siRNA**	**Sequence (5’ → 3’)**	**Position in sequence**	**GenBank Accession #**
STAT1-siRNA			
Sense	GCGUAAUCUUCAGGAUAAUtt	nt 570-590	NM_001384888.1
Antisense	AUUAUCCUGAAGAUUACGCtt		
CREB1-siRNA			
Sense	GAGAGAGGUCCGUCUAAUGtt	nt 994-1012	NM_004379.5
Antisense	CAUUAGACGGACCUCUCUCtt		
Control-siRNA			
Sense	UUCUCCGAACGUGUCACGUtt	Scramble	
Antisense	ACGUGACACGUUCGGAGAAtt		

Table S3. List of the top 100 genes correlated with STAT1 in pan-cancer.

Gene name	Ensembl ID	R score
APOL6	ENSG00000221963	0.949179
GBP1	ENSG00000117228	0.933621
XRCC5	ENSG00000079246	0.924181
TRIM69	ENSG00000185880	0.917219
BTN3A3	ENSG00000111801	0.91643
GBP1P1	ENSG00000225492	0.915426
GBP4	ENSG00000162654	0.910979
ICOS	ENSG00000163600	0.909393
GBP5	ENSG00000154451	0.903139
TAP1	ENSG00000168394	0.901463
EPSTI1	ENSG00000133106	0.898595
AIM2	ENSG00000163568	0.89777
CXCL10	ENSG00000169245	0.894202
CTLA4	ENSG00000163599	0.892353
GCC2	ENSG00000135968	0.892067
TAP2	ENSG00000204267	0.890167
CXCL9	ENSG00000138755	0.889873
IRF1	ENSG00000125347	0.88917
PARP9	ENSG00000138496	0.888576
P2RY10	ENSG00000078589	0.888396
TLR8	ENSG00000101916	0.887592
HAT1	ENSG00000128708	0.884931
GPN1	ENSG00000198522	0.88466
BZW1	ENSG00000082153	0.884223
YWHAQ	ENSG00000134308	0.88388
BTN3A1	ENSG00000026950	0.882993
SF3B1	ENSG00000115524	0.882537
B2M	ENSG00000166710	0.882492
PTPRC	ENSG00000081237	0.881562
EIF2AK2	ENSG00000055332	0.880478
MMADHC	ENSG00000168288	0.879902
TXNDC9	ENSG00000115514	0.87936
SP140	ENSG00000079263	0.879253
*	ENSG00000226751	0.878028
SLC30A6	ENSG00000152683	0.877431
CSF2RB	ENSG00000100368	0.876883
USP37	ENSG00000135913	0.876364
CD38	ENSG00000004468	0.876275
SEC63	ENSG00000025796	0.875037
PDIA6	ENSG00000143870	0.874928
ARPC2	ENSG00000163466	0.874838
SAMSN1	ENSG00000155307	0.874808

SRBD1	ENSG00000068784	0.873909
PIGK	ENSG00000142892	0.873036
APOL3	ENSG00000128284	0.873022
TNFAIP8	ENSG00000145779	0.872863
GIMAP4	ENSG00000133574	0.872534
RO60	ENSG00000116747	0.871651
IFNG	ENSG00000111537	0.871337
FBXO11	ENSG00000138081	0.870586
UBD	ENSG00000213886	0.870362
PARP14	ENSG00000173193	0.870192
CEBPZ	ENSG00000115816	0.870139
SEPTIN2	ENSG00000168385	0.870022
CCR5	ENSG00000160791	0.868923
STAT3	ENSG00000168610	0.867776
TSN	ENSG00000211460	0.867731
FASLG	ENSG00000117560	0.867447
BCLAF1	ENSG00000029363	0.866774
FYB1	ENSG00000082074	0.866554
ACTR2	ENSG00000138071	0.865832
HLA-F	ENSG00000204642	0.865051
P2RX7	ENSG00000089041	0.864765
HNRNPLL	ENSG00000143889	0.864443
GPR171	ENSG00000174946	0.864321
CTSS	ENSG00000163131	0.864288
FCRL3	ENSG00000160856	0.863874
JAK1	ENSG00000162434	0.863339
WDFY1	ENSG00000085449	0.863272
IRF8	ENSG00000140968	0.86321
TRIP12	ENSG00000153827	0.862919
CREB1	ENSG00000118260	0.862914
BTLA	ENSG00000186265	0.862555
ATF6	ENSG00000118217	0.862161
APOL2	ENSG00000128335	0.861876
LANCL1	ENSG00000115365	0.861744
GIMAP7	ENSG00000179144	0.861334
NLRC5	ENSG00000140853	0.860839
DDX60	ENSG00000137628	0.86068
XAF1	ENSG00000132530	0.860357
LCP2	ENSG00000043462	0.860065
CDC73	ENSG00000134371	0.859906
RAB1A	ENSG00000138069	0.859701
CUL3	ENSG00000036257	0.85951
DYNC1I2	ENSG00000077380	0.859281
SAMD9L	ENSG00000177409	0.859174

FCGR3A	ENSG00000203747	0.859137
PAPOLG	ENSG00000115421	0.859097
RASSF5	ENSG00000266094	0.859009
DHX40	ENSG00000108406	0.85857
AL645939.2	ENSG00000225864	0.858249
AGPS	ENSG00000018510	0.857958
SLC4A1AP	ENSG00000163798	0.85793
TNFSF13B	ENSG00000102524	0.857655
STAMBP	ENSG00000124356	0.857645
DHX9	ENSG00000135829	0.856498
NOD2	ENSG00000167207	0.856382
OAS2	ENSG00000111335	0.856074
PTPN22	ENSG00000134242	0.855639

*, no gene name.

Table S4. The STAT1 binding score of the top 100 genes sorted by k_{max}.

Gene name	Ensembl ID	k_{total}	k_{max1}	k_{max3}
AL157385.1	ENSG00000228667	160.068	22.53219	48.52235
AC100861.2	ENSG00000250714	284.608	22.53219	46.20313
AL137186.2	ENSG00000232807	138.9717	22.53219	41.84774
EIF2AK2	ENSG00000055332	199.1241	22.11564	43.55253
ZIC1	ENSG00000152977	119.7169	22.11564	47.56548
C8A	ENSG00000157131	113.3239	21.97269	44.49495
AC012531.6	ENSG00000277129	104.6659	21.97269	43.47107
AC012531.4	ENSG00000274817	119.3996	21.97269	44.80573
AC012531.7	ENSG00000277994	85.80158	21.97269	42.15842
OAS2	ENSG00000111335	159.7829	21.95081	56.67962
USP12P2	ENSG00000224007	220.1232	21.95081	46.38777
STX6	ENSG00000135823	91.42209	21.91618	38.29513
TRBV15	ENSG00000276819	142.3015	21.91618	38.54818
SNORD114-6	ENSG00000201263	118.3208	21.59858	49.92391
SNORD114-5	ENSG00000199798	82.17477	21.59858	40.08953
MEG8	ENSG00000225746	115.3342	21.59858	49.92391
AL132709.1	ENSG00000200150	89.77864	21.59858	40.08953
SAMM50P1	ENSG00000224488	76.44856	21.59858	41.4868
SNORD114-4	ENSG00000200832	57.57875	21.59858	34.9224
AC087501.3	ENSG00000263051	129.1909	21.59858	43.83975
PYCARD	ENSG00000103490	48.10225	21.59858	38.77635
SAMD9L	ENSG00000177409	187.9084	21.55615	51.35749
RNU6-225P	ENSG00000207343	211.5002	21.41511	50.17518
EIF3M	ENSG00000149100	171.0251	21.41511	41.53675
AC008957.1	ENSG00000250155	103.496	21.41511	43.14126
OAZ1	ENSG00000104904	137.8494	21.33481	48.55417
AL020995.2	ENSG00000276645	166.8632	21.33481	47.36205
AC005258.1	ENSG00000273734	137.8494	21.33481	48.55417
AL160163.2	ENSG00000227516	122.9497	21.16897	32.36465
RPAP2P1	ENSG00000270874	180.891	21.16897	45.81053
PSMA2P1	ENSG00000254582	234.6033	21.15472	45.9454
GNL3LP1	ENSG00000215032	118.2826	21.08119	44.09509
RNA5SP173	ENSG00000201727	128.4596	21.08119	40.62825
CADM3	ENSG00000162706	111.5444	21.08119	40.44088
AP000873.4	ENSG00000254676	191.8755	21.03909	47.87265
AC108734.3	ENSG00000242068	162.5918	21.03909	43.94662
AP001496.1	ENSG00000264254	57.60134	21.03909	37.8099
RAP1GAP2	ENSG00000132359	179.2363	21.02364	40.40288
AC093249.5	ENSG00000261680	98.48809	21.01721	36.78256
AC098862.1	ENSG00000250611	92.8048	21.01721	43.98197
AC093249.1	ENSG00000260113	118.5676	21.01721	40.14686
AC008080.3	ENSG00000233219	165.4575	21.01721	47.27143

AC117456.1	ENSG00000200616	275.5456	21.01721	44.09576
LINC01239	ENSG00000234840	134.5068	21.01721	41.63804
AC025062.2	ENSG00000271654	133.0432	20.98258	43.60845
RPL34P26	ENSG00000223676	102.1925	20.98258	46.88962
EPHA3	ENSG00000044524	259.7176	20.87569	44.09524
AC009227.2	ENSG00000238004	175.1022	20.87569	41.77947
IMPA1	ENSG00000133731	48.27565	20.87569	38.67612
AC111194.1	ENSG00000248317	123.9937	20.87569	40.12347
AC009227.1	ENSG00000224675	163.2677	20.87569	40.00586
IFIT1	ENSG00000185745	204.34	20.85562	44.32459
THRB-IT1	ENSG00000224822	197.3734	20.80277	41.69125
RNU1-32P	ENSG00000200807	197.1736	20.79911	46.96266
AC005674.2	ENSG00000261490	93.33505	20.76936	37.48588
USP6	ENSG00000129204	124.0654	20.76936	40.60159
EPB42	ENSG00000166947	118.7067	20.76936	38.92804
AC092916.2	ENSG00000240787	80.87658	20.76936	39.21314
TRMT112P5	ENSG00000250161	230.6988	20.74626	49.40236
RN7SL89P	ENSG00000243383	95.70823	20.74626	49.00582
AC019064.1	ENSG00000237320	99.86215	20.74626	37.25553
AC244100.3	ENSG00000275944	157.2153	20.60948	51.94833
AC009948.4	ENSG00000279598	187.1054	20.60948	45.7667
HMGN1P26	ENSG00000259557	139.6266	20.60948	44.96627
ZNF177	ENSG00000188629	336.3849	20.60948	46.60673
MRRF	ENSG00000148187	158.9561	20.60948	46.60463
LCMT1	ENSG00000205629	183.1578	20.60948	40.0367
AC244100.4	ENSG00000278690	149.3642	20.60948	51.94833
RBM18	ENSG00000119446	161.0025	20.60948	46.60463
CBLL2	ENSG00000175809	199.3349	20.58759	40.62141
SIGLEC1	ENSG00000088827	32.36738	20.58759	30.16033
AC024579.1	ENSG00000251044	130.0653	20.57334	46.2491
AP001636.1	ENSG00000254786	76.96168	20.57334	35.73268
ANKRD62P1	ENSG00000259271	332.8172	20.55297	50.49097
AL161896.1	ENSG00000269599	143.0045	20.55297	46.05681
AC012442.1	ENSG00000207383	108.4485	20.53872	38.28265
FOSB	ENSG00000125740	86.07786	20.53872	44.51069
N4BP1	ENSG00000102921	90.70082	20.49981	45.93074
ARNTL	ENSG00000133794	115.0998	20.49981	38.83891
TRMT61A	ENSG00000166166	80.46273	20.49981	46.86867
IFI44	ENSG00000137965	132.1183	20.49981	51.00682
AC084880.1	ENSG00000213144	110.5145	20.49981	45.19719
AL391099.1	ENSG00000279239	111.6385	20.49981	49.54137
AL133367.1	ENSG00000260285	80.46273	20.49981	46.86867
JKAMP	ENSG00000050130	115.9886	20.49981	49.07956
YAP1P2	ENSG00000236080	134.4372	20.49981	45.98448

CCNYL2	ENSG00000182632	122.9545	20.49981	47.38278
L3HYPDH	ENSG00000126790	117.602	20.49981	49.07956
AC090142.2	ENSG00000243939	185.5429	20.49981	44.37786
LINC00951	ENSG00000204092	70.89773	20.49981	39.72203
MIR6505	ENSG00000275770	183.3795	20.49981	43.57186
MAX	ENSG00000125952	91.95089	20.48151	38.57765
LARS2	ENSG00000011376	193.4262	20.48151	42.12569
AC009163.3	ENSG00000260092	192.9636	20.46414	45.86702
OR10G5P	ENSG00000226461	98.05483	20.46414	39.05272
TNRC18	ENSG00000182095	63.53427	20.46414	32.15312
OR10G4	ENSG00000254737	54.58933	20.46414	30.16463
CYBB	ENSG00000165168	174.4067	20.46414	46.49808
TRPM6	ENSG00000119121	112.0184	20.46414	45.16142
NOL8P1	ENSG00000248327	303.2002	20.46414	50.83105
