# Supplementary material for: Target Finder of Transcription Factor (TFoTF): a novel tool to predict transcription factor‐targeted genes in cancer
Source: Mol Oncol. 2023 Feb 11;17(7):1246–62. doi: 10.1002/1878-0261.13388 (PMC10323881; doi:10.1002/1878-0261.13388)
Supplement: Supplementary file 2 — Appendix S1. Tutorial for TFoTF. [file MOL2-17-1246-s002.pdf]

Tutorial for TFoTF

Contents

Introduction of the required files for TFoTF execution 1
How to build the computing platform required for TFoTF 2
Execution steps of TFoTF 3
How to get a PWM data file 6
System Information 8

Introduction of the required files for TFoTF execution

After downloading and unpacking, you can get a "sample" folder. The internal structure of the "sample" folder is shown in Figure 1.

Figure 1

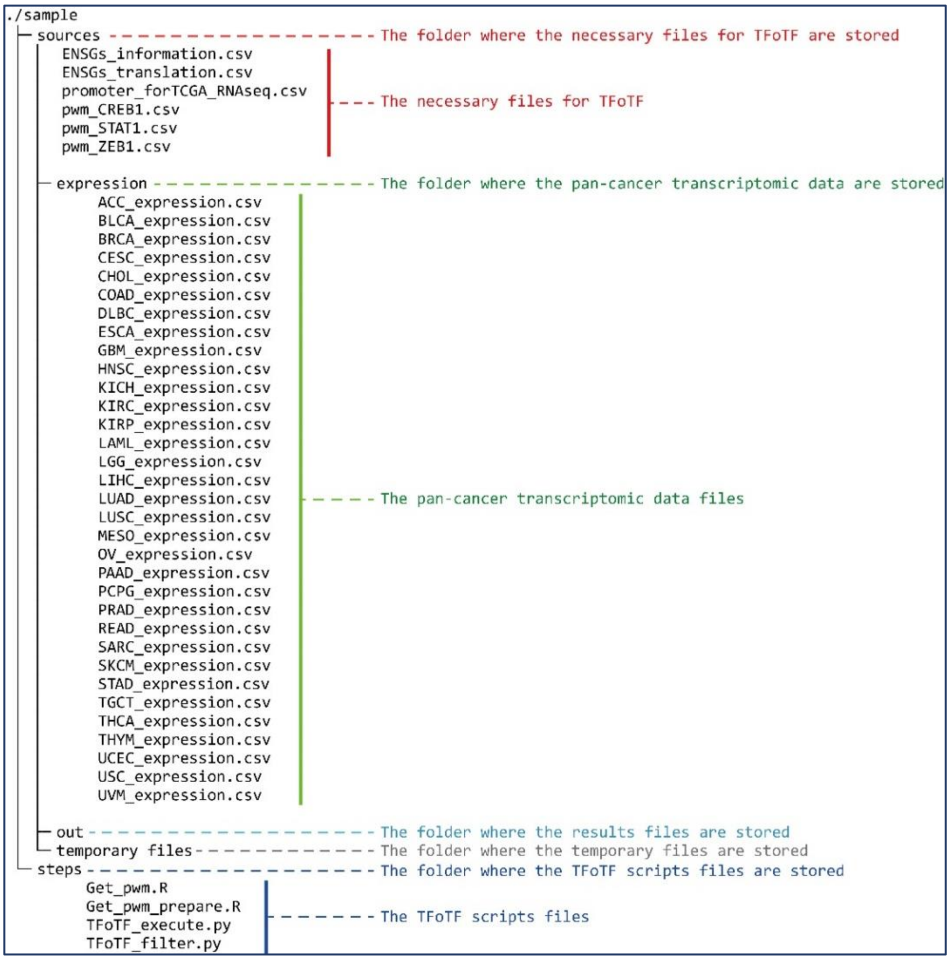

How to build the computing platform required for TFoTF

a) Install and set up the python 3 runtime environment. We recommend using anaconda, as it already integrates the required environment for TFoTF. You can download the anaconda distribution from its website (https://www.anaconda.com/products/distribution) and follow the instructions to install it.

b) After finishing the installation, you can find anaconda3 in the start menu (Figure 2). Click to open the Spyder, which is the IDLE (Integrated Development and Learning Environment) you need to run TFoTF. Alternatively, if you have computer-related experience, you can also use other ways to build the runtime environment and use other IDLEs as you like.

Figure 2

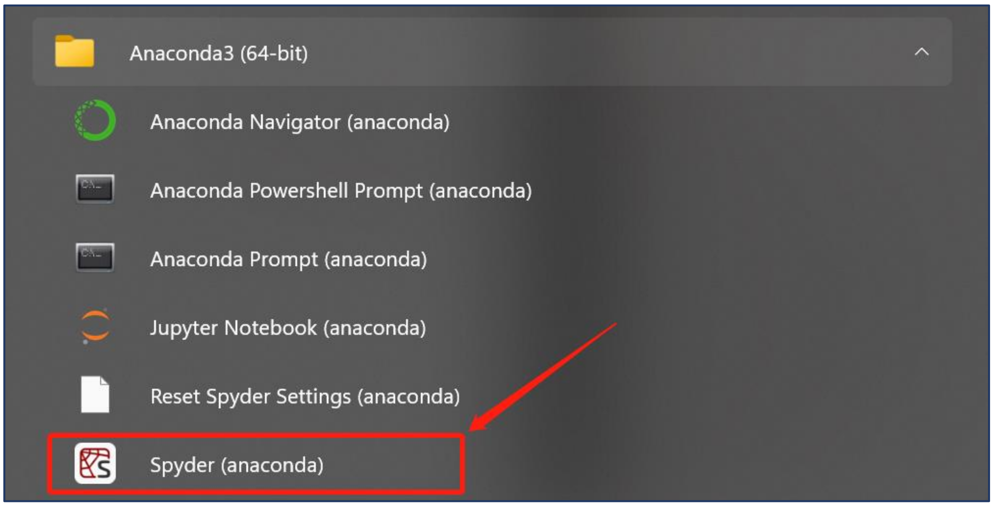

Execution steps of TFoTF

a) Open “./sample/steps/TFoTF_execute.py” in Spyder.

b) After opening “TFoTF_execute.py”, you will see the screen shown in Figure 3, where you can set the relevant operating parameters of TFoTF.

Figure 3

```
# step 1: Set workdir, for example, 'F:/sample/sources'.
set_dir = 'E:/sample/sources'

# step 2: Set target TF.
# You must have the corresponding pwm data file, like './sample/sources/pwm_STAT1.csv'. Use uppercase letters.
target_gene = 'STAT1'

# step 3: Set cut-offs to determine the significance.
pvalue_cut = 0.05 # from 0 to 1

# step4: Run this script.
```

set_dir: set the working directory (You must set it by yourself)

target_gene: the gene name of the transcription factor you want to predict (You must have the corresponding PWM data file, like “./sample/sources/pwm_STAT1.csv”. Refer to section “How to get a PWM data file”)

pvalue_cut: set the significance p-value of the correlation analysis (recommended to keep the default, i.e., 0.05)

NOTICE: If you are not sure about the meaning of "directory" in computer, we recommend you refer to Wikipedia (https://en.wikipedia.org/wiki/Directory_(computing)) for basic concepts. When setting the directory, please note that you should use a slash (/), rather than a backslash (\). In directories, backslashes are used in windows, whereas slashes are used in python. You must always be careful not to misuse them. For example, “E:/sample/sources” is the right directory, while "E:\sample\sources" is wrong, in python.

c) After completing the above three settings, click on the green triangle in the upper toolbar to run the script (Figure 4). Because TFoTF is a genome-wide target gene prediction tool. The whole computing process takes several hours to more than ten hours. The exact computational time depends on your computer hardware.

Figure 4

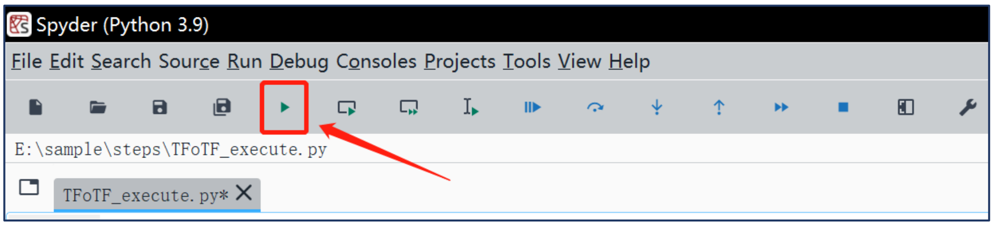

d) After whole calculations are completed, the two results file is saved in "./sample/sources/out" (Figure 5).

Figure 5

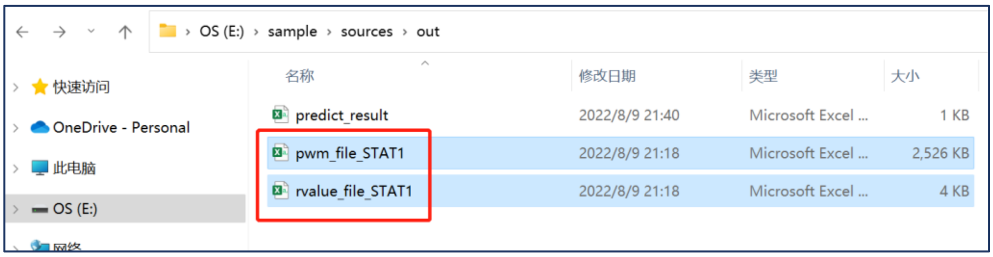

e) You can open "./sample/steps/TFoTF_filter.py" in Spyder, and set cut-offs according to your needs to get the predicted gene list (Figure 6).

Figure 6

```
# set workdir, for example, 'F:/sample/sources/out'
set_dir = 'E:/sample/sources/out'

# Set the pearson correlation R score cut-off, Recommend > 0.6
rvalue_cutoff = 0.6

# Set the type of pwm score, you can choose between 'max1', 'max3', 'total'
pwm_type = 'max1'

# Set the pwm score cut-off, Recommended > 8 for max1
pwm_cutoff = 8

# The previously calculated processed data
rvalue_file = 'rvalue_file_STAT1.csv'
pwm_file = 'pwm_file_STAT1.csv'

# name the result file, and the file will be created in your workdir
savefile_to = 'predict_result.csv'
```

set_dir: set the working directory (You must set it by yourself)

rvalue_cutoff: set the R score cut-off (We recommended keeping the default, i.e., 0.6)

pwm_type: choose the type of PWM score (We recommended keeping the default, i.e., max1)

pwm_cutoff: set the PWM score cut-off (We recommended keeping the default, i.e., 8)

rvalue_file: The name of the previously calculated “rvalue_file” data (You must set it by yourself)

pwm_file: The name of the previously calculated “pwm_file” data (You must set it by yourself)

savefile_to: name the result file, and the file will be created in your working directory (You must set it by yourself).

f) Click on the green triangle in the upper toolbar to run the script (Figure 4), and the predicted target genes list (predict_result) will be saved in your working directory (Figure 7).

Figure 7

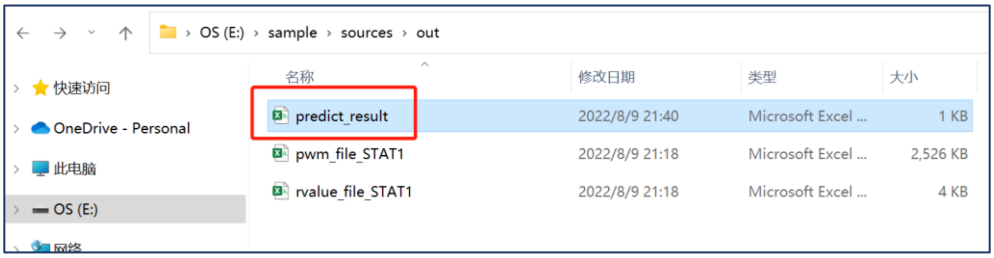

How to get a PWM data file

a) Install and set up the R runtime environment. We recommend using R studio. You can download and install R from https://www.r-project.org/; download and install R studio from https://www.rstudio.com/.

b) Open and run "./sample/steps/Get_pwm_prepare.R" in R studio to install dependent packages.

c) You can use the website JASPAR (https://jaspar.genereg.net/) to search for the gene name of the transcription factor and get the corresponding JASPAR ID (Figure 8), like CREB1: MA0018.4 (Figure 9).

Figure 8

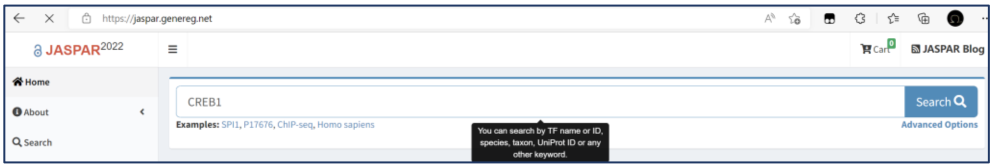

Figure 9

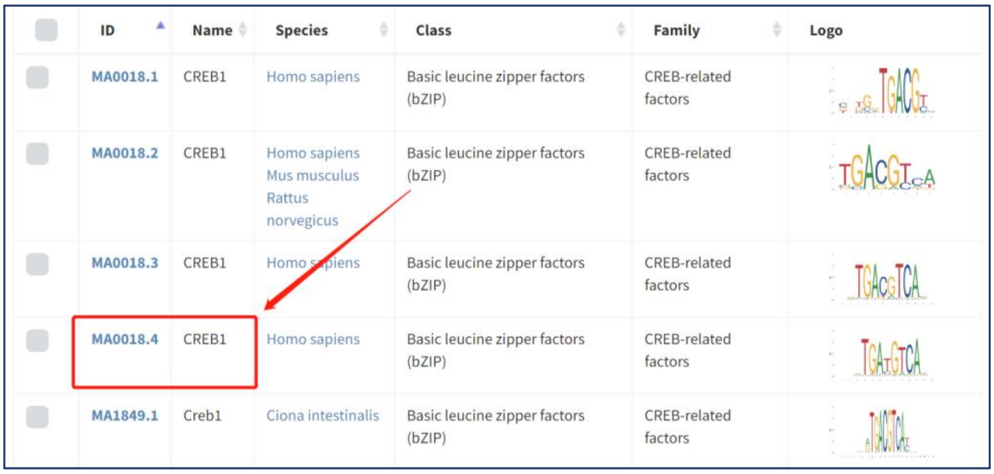

	ID	Name	Species	Class	Family	Logo
	MA0018.1	CREB1	Homo sapiens	Basic leucine zipper factors (bZIP)	CREB-related factors	
	MA0018.2	CREB1	Homo sapiens Mus musculus Rattus norvegicus	Basic leucine zipper factors (bZIP)	CREB-related factors	
	MA0018.3	CREB1	Homo sapiens	Basic leucine zipper factors (bZIP)	CREB-related factors	
	MA0018.4	CREB1	Homo sapiens	Basic leucine zipper factors (bZIP)	CREB-related factors	
	MA1849.1	Creb1	Ciona intestinalis	Basic leucine zipper factors (bZIP)	CREB-related factors	

d) Open "./sample/steps/Get_pwm.R" in R studio, input the corresponding JASPAR ID in "ID = " and set the output directory in "output = ", as shown in Figure 9, then run this script to get the PWM data corresponding to the transcription factor. The obtained PWM data file should be stored in the "./sample/sources" folder, and please name the PWM data file to follow the rules of "pwm_'name of TF'.csv" as shown in Figure 10.

Figure 10

Get_pwm.R*

```r
library(Biostrings)
library(TFBSTools)
library(JASPAR2020)

ID = 'MA0018.4'
output = 'E:/sample/sources/pwm_CREB1.csv'

pfm<-getMatrixByID(JASPAR2020,ID)
pwm<-toPWM(pfm)
write.csv(pwm@profileMatrix, output)
```

System Information

Operating System: Windows 11 Home Edition Version 21H2
Anaconda: ver. 2.1.4
Spyder: ver 5.1.5
R: ver 4.2.1
R studio: RStudio 2022.07.1+554 "Spotted Wakerobin" Release for windows
